# Synthesis and Characterization of Camphorimine Au(I) Complexes with a Remarkably High Antibacterial Activity towards *B. contaminans* and *P. aeruginosa*

**DOI:** 10.3390/antibiotics10101272

**Published:** 2021-10-19

**Authors:** Joana P. Costa, Sílvia A. Sousa, Catarina Soeiro, Jorge H. Leitão, Adelino M. Galvão, Fernanda Marques, M. Fernanda N. N. Carvalho

**Affiliations:** 1 Centro de Química Estrutural e Departamento de Engenharia Química, Instituto Superior Técnico, Universidade de Lisboa, Av. Rovisco Pais, 1049-001 Lisboa, Portugal; joanavcosta@tecnico.ulisboa.pt (J.P.C.); adelino@tecnico.ulisboa.pt (A.M.G.); 2 Department of Bioengineering, iBB-Institute of Bioengineering and Biosciences, Instituto Superior Técnico, University of Lisbon, 1049-001 Lisbon, Portugal; sousasilvia@tecnico.ulisboa.pt (S.A.S.); catarina.soeiro25@gmail.com (C.S.); 3 Associate Laboratory, i4HB—Institute for Health and Bioeconomy at Instituto Superior Técnico, Universidade de Lisboa, Av. Rovisco Pais, 1049-001 Lisboa, Portugal; 4 Departamento de Engenharia e Ciências Nucleares, Instituto Superior Técnico, University of Lisbon, 2695-066 Bobadela, Portugal; fmarujo@ctn.tecnico.ulisboa.pt

**Keywords:** gold complexes, camphorimines, antibacterial properties, *B. contaminans*, *P. aeruginosa*, gold content/antimicrobial activity correlations

## Abstract

Fourteen new camphorimine Au(I) complexes were synthesized and characterized by spectroscopic (NMR, FTIR) and elemental analysis. The structural arrangement of three selected examples were computed by Density Functional Theory (DFT) showing that the complexes essentially keep the {Au^I^-CN} unit. The Minimum Inhibition Concentrations (MIC) were assessed for all complexes showing that they are active towards the Gram-negative strains *E. coli* ATCC25922, *P. aeruginosa* 477, and *B. contaminans* IST408 and the Gram-positive strain *S. aureus* Newman. The complexes display very high activity towards *P. aeruginosa* 477 and *B. contaminans* IST408 with selectivity towards *B. contaminans*. An inverse correlation between the MIC values and the gold content was found for *B. contaminans* and *P. aeruginosa*. However, plots of MIC values and Au content for *P. aeruginosa* 477 and *B. contaminans* IST408 follow distinct trends. No clear relationship could be established between the MIC values and the redox potentials of the complexes measured by cyclic voltammetry. The MIC values are essentially independent of the redox potentials either cathodic or anodic. The complexes K_3_[{Au(CN)_2_}_3_(^A4^L)] (**8**, Y = m-OHC_6_H_4_) and K_3_[{Au(CN)_2_}_3_(^B2^L)]·3H_2_O (**14**, Z = p-C_6_H_4_) display the lower MIC values for the two strains. In normal fibroblast cells, the IC_50_ values for the complexes are ca. one order of magnitude lower than their MIC values, although higher than that of the precursor KAu(CN)_2_.

## 1. Introduction

The medicinal properties of camphor have been recognized since ancient times, having a long history of traditional applications. Pharmacological uses of camphor include liniments and balms for relief of muscular pain, inhalants for nasal decongestion, antitussives, and expectorants. The activity of camphor on nasal decongesting was attributed to the stimulation of cold receptors in the nose [1,2,3]. Such behavior triggered our interest in the ability of camphor derivatives, in particular camphor derived complexes, to interact with other biological targets and also to evaluate their antibacterial properties. With that purpose, we prepared silver and copper complexes and investigated their antimicrobial and cytotoxic properties. The results obtained so far show that several silver camphorimine complexes have remarkable antimicrobial activities [4,5,6,7], which in a few cases combine with their anticancer properties [8]. The design of the steric and redox characteristics of the metal sites [9] allow to tune their activity and selectivity as confirmed by replacement of nitrate by hydroxy at the coordination sphere of Ag(I), which switched the activity of the complexes from no-active to highly active towards *Candida albicans* [6].

In complexes, the metal plays a relevant role in the biological activity, as evidenced by studies on silver based camphorimine complexes that showed as very efficient antifungals [4] and moderate to high antibacterials [5,7,9] with additional high cytotoxic activities [8]. No such behavior was found for the related copper camphorimine complexes that just showed moderate cytotoxic activity [10]. The role of the camphorimine ligands, which typically do not display any biological activity, is to tune the properties of the complexes for biological applications. To fulfil that purpose, the camphorimine compounds are particularly attractive because they are easy to synthesize and allow the design of molecules with considerably distinct steric and electronic properties, in order to enable interaction with specific targets in the cell.

Aiming to improve the results obtained towards bacteria with silver complexes, we decided to synthesize gold camphorimine complexes and study their antibacterial properties. Gold was selected, taking in consideration that it belongs to the same group of silver and copper (Periodic table, group 11) and has medicinal uses that date back to ancient cultures, such as those of India and Egypt [11]. The reason to choose potassium gold dicyanide (KAu(CN)_2_) as gold precursor was that it is ready available and was reported as being used to treat tuberculosis [11], therefore, accepted as a pharmaceutical agent. Recent studies on cationic gold-based compounds (Au(I) and Au(III)) showed that they are promising as antimicrobial and anticancer agents [12]. The reference drug Auranofin, approved for the treatment of rheumatoid arthritis, is currently under study for therapeutic applications such as cancer, and bacterial and parasitic infections [13].

Although the mechanisms of gold biological activities remain largely unknown, evidence suggests that Au(I) is selective for enzymes with sulfhydryl or selenol groups. Compounds derived from either Au(I) or Au(III) have been pointed out as inhibitors of thioredoxin reductase (TrxR), affecting the cell thiol-redox homeostasis [14]. In what concerns the cytotoxic properties, Au(III) complexes seem to exhibit activities similar to those of the isoelectronic Pt(II) in the Cisplatin complex [15,16,17].

The herein results aim at contributing to face the bacteria antimicrobials resistance threat, based on gold derived complexes with antibacterial activities. For that purpose, a set of Au(I) camphorimine complexes was synthesized and their antibacterial activities evaluated against the Gram-negative strains *E. coli* ATCC25922, *P. aeruginosa* 477, and *B. contaminans* IST408, and the Gram-positive *S. aureus* Newman.

To the best of our knowledge, this is the first report on the synthesis and characterization of this type of gold complexes. The newly synthesized Au(I) camphorimine complexes were chemically characterized and their antimicrobial properties assessed, showing a particularly high activity against the Gram-negative *B. contaminans* and *P. aeruginosa*.

## 2. Results

### 2.1. Synthesis and Characterization

**Scheme 1:**
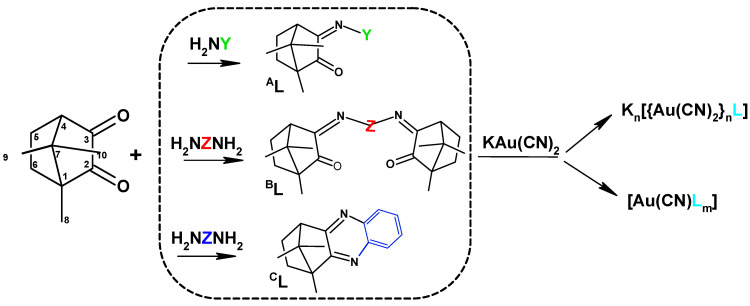
Scheme for synthesis of the Au(I) complexes from camphorimine (^A^L), bi-camphor (^B^L), or camphor phenazine (^C^L) types.

Camphor derivatives of the imine type (^A^L, OC_10_H_14_NY) were obtained by condensation of amines or hydrazines with camphor quinone, while from condensation of *para* or *orto* diamines, bi-camphor (^B^L, OC_10_H_14_N)_2_Z) or camphor phenazine (^C^L, NC_10_H_14_NY) type species were, respectively, obtained (Scheme 1).

By reaction of the camphor derivatives (^A^L, ^B^L or ^C^L) with potassium gold dicyanide (KAu(CN)_2_), two main types of complexes were obtained: those that kept the KAu(CN)_2_ unit and those that lost the KCN moiety to afford the Au(CN) unit center (Scheme 1).

The anionic [Au(CN)_2_]^−^ unit is known to form coordination polymers acting as a spacer between gold and cationic Cu(II), Zn(II), Ni(II), Co(II), Sn(II) metal centers [18,19,20]. Since the camphor ligands are neutral, no such type of interaction is feasible. However, adducts with a variety of metal to ligand ratios [{KAu(CN)_2_}_n_(L)] (*n* = 1, 3, 5, 7) were reproductively obtained. A tentative to rationalize such a reactivity trend is based on the interaction of the potassium ion with several nitrogen atoms of the neighbor molecules as found for the precursor potassium gold dicyanide by X-ray diffraction analysis. Potassium gold dicyanide displays a polynuclear tridimensional structure formed by alternating linear anionic (Au(CN)_2_^−^) and cationic (K^+^) layers, with each potassium ion interacting with several nitrogen atom of distinct Au(CN)_2_ units [21].

Although the camphorimine gold adducts with nuclearity higher than three are intriguing, they will be considered as dopped gold potassium dicyanide species and, therefore, they are not further discussed or their biological properties studied.

Depending on the characteristics of the ligand (^A^L, ^B^L) and the experimental conditions, release of KCN affords complexes of formula [Au(CN)L] (L = ^A^L, ^B^L) [Au(CN)L_n_] (L = ^A^L, ^B^L; n = 2,3), [{Au(CN)}_2_(^A^L)]. No such type of complexes was obtained for ligand ^C^L. As far as we know, formation of {Au(CN)} from potassium gold dicyanide was not reported before.

All complexes were formulated based on analytical and spectroscopic properties (See Experimental). In order to elucidate the structural arrangement and geometry of the complexes, computational calculations by DFT were undertaken, since no suitable crystals could be obtained to perform single crystal X-ray diffraction analysis.

### 2.2. Computational Calculations

**Figure 1:**
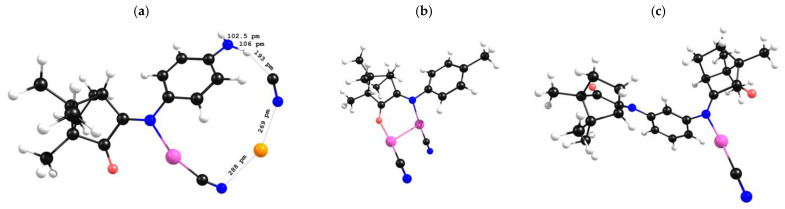
DFT optimized structure for complexes: (**a**) **1**, (**b**) **6,** and (**c**) **13**.

DFT calculations were carried out using GAMESS-US [22] version R3 with a B3LYP functional, using a SBKJC basis set. Structures for the selected complexes K[Au(CN)_2_(^A1^L)] (**1**, Y = C_6_H_4_NH_2_), [{Au(CN)}_2_(^A2^L)] (**6**, Y = C_6_H_4_CH_3_), and [Au(CN)(^B1^L)] (**13**, Z = *m*-C_6_H_4_) converged to the structural arrangements displayed below (Figure 1). The optimized structures were confirmed as minimums by Hessians with positive eigenvalues and six near zero frequencies.

Structures with one and two cyanide groups *per* gold atom were attempted. However, all the essayed structures with the fragment {Au(CN)_2_} underwent dissociative pathways releasing the ligand and regenerating the [Au(CN)_2_]^−^ unit. All the structures converged to [Au(CN)L] in a linear arrangement (see complex **13**, Figure 1). In some cases, the released KCN can co-crystallize (**1**, Figure 1) or a second unit of AuCN is incorporated (complex **6** in Figure 1). The second gold cyanide unit binds to the {Au(CN)L} fragment through a week Au-Au bond (bond order 0.178) as well as a week Au-O bond (bond order 0.234). Although stronger, the Au-N bond is very labile with a bond order of 0.324.

### 2.3. Antibacterial Activity

The antibacterial properties of the camphorimine Au(I) complexes were assessed experimentally through the determination of the Minimum Inhibitory Concentration (MIC) against the Gram-positive strain *S. aureus* Newman and the Gram-negative strains *E. coli* ATCC25922, *P. aeruginosa* 477, and *B. contaminans* IST408. Experimental results show that all the complexes are active towards the bacterial strains under evaluation. As a general trend, complexes perform better for *P. aeruginosa* 477 and *B. contaminans* IST408 than for *E. coli* ATCC25922 or *S. aureus* Newman, although complexes **1**, **2,** and **10** display a reasonable activity against all the strains under study (Table 1).

As previously reported, the ligands are not active against the upper mentioned strains [9].

**Figure 2:**
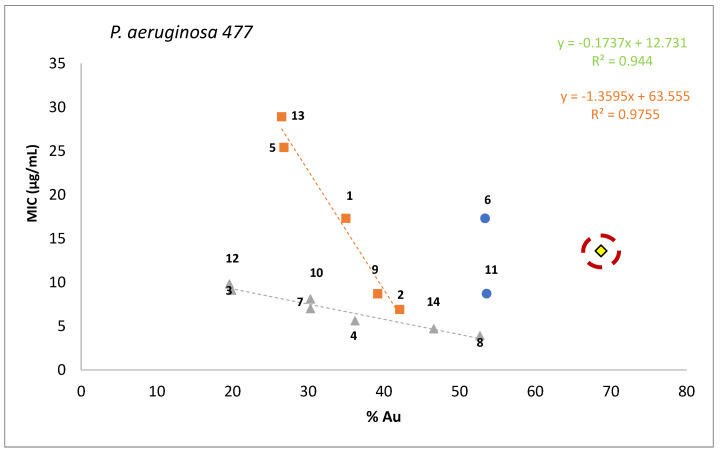
Relationship between gold content (% Au) of complexes and their MIC values against *P. aeruginosa* (numbers close to the marks refer to the number of the complexes, see Table 1).

**Figure 3:**
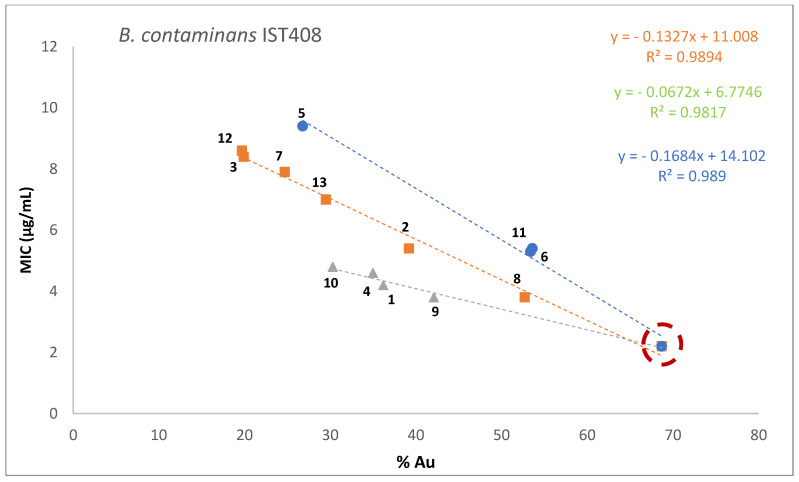
Relationship between gold content of complexes and their MIC values against *B. contaminans* (numbers close to the marks refer to the number of the complexes, see Table 1).

In order to try to correlate the characteristics (metal content, ionic/neutral character, nuclearity) of the complexes with their antibacterial activity, the MIC values obtained for *P. aeruginosa* 477 and *B. contaminans* IST408 (Table 1) were graphically depicted versus the metal content of each complex (Figure 2 and Figure 3).

Plots in Figure 2 and Figure 3 clearly evidence a direct relationship between the antibacterial activities of the complexes and their gold content for the *B. contaminans* and *P. aeruginosa* strains, i.e., the higher the gold content, the higher the activity, the lower the MIC values. However, the correlation pattern is considerably different for the two strains. While for *P. aeruginosa* 477, the straight lines fitting the points established by the MIC value and the Au content do not cross the point defined by values for the potassium gold dicyanide precursor (Figure 2), for *B. contaminans* IST408 all lines converge to the point defined by the MIC/Au values for KAu(CN)_2_ (Figure 3). Such trend suggests that the gold cyanide unit is the key factor driving the activity towards *B. contaminans*, no matter if the complexes have an anionic [Au(CN)_2_L]^−^ or neutral [Au(CN)L] character, although the ionic character ([Au(CN)_2_L]^−^) prompts better results (lower MIC values). The amine and hydroxy substituent at the camphorimine ligands (**1**, Y = C_6_H_4_NH_2_; **9**, Y = NH_2_; **10**, Y = OH) further enhance the antibacterial activity of the ionic complexes K[Au(CN)_2_L], conceivably due to the facile hydrogen interactions with cell components. Such benefit is lost for the neutral complex [Au(CN)(**^A1^**L_3_)]·H_2_O (**3**, Y = C_6_H_4_NH_2_) with low gold content. That is not the case for *P. aeruginosa* for which complex **3** displays one of the lowest MIC values (Figure 2). Such trend evidence that different mechanisms of action operate for the two bacterial species. In fact, the plots defined for *P. aeruginosa* (Figure 2) stay away from the point defined by the gold cyanide precursor. In this case, the gold cyanide content remains relevant but structural effects due to the gold cyanide unit {Au(CN)_2_^−^} seem less important than for *B. contaminans*. In the case of complexes with bi-camphor ligands, symmetry conceivably play a role in the activity, since the *para* spacer at complex **14** considerably enhances the antibacterial activity compared to the *meta* spacer at complex **13** (Figure 2). The high symmetry of the *para* bicamphor ligand [23] rends almost equally accessible four binding atoms (N, O) to interaction with bacteria receptors.

The effect of the nuclearity, number, and eleFctronic characteristics of the ligands at the antibacterial properties of the complexes does not show a trend. However, it is evident that the ligands fine-tune the antibacterial activity, according to the slope of the lines (Figure 2 and Figure 3).

The distinct trend for the activity/gold content observed for the two bacterial strains (*B. contaminans* and *P. aeruginosa*) cannot be attributed to the cell wall structural arrangement, since both species are Gram-negative, with a complex cell wall composed of an inner and an outer membrane.

### 2.4. Toxicity

The assessment of the cytotoxicity of new complexes is an essential step in the investigation of their application as prospective drugs. The first screenings are in vitro studies using normal cells such as human fibroblasts. The MTT assay was selected to assess cell viability by measuring the activity of a mitochondrial succinate dehydrogenase as the end-point of cytotoxicity [24,25]. Results obtained with the gold camphorimine complexes indicated that in general they have high cytotoxicity presenting IC_50_ values in the range 0.15–0.40 µg/mL. Such cytotoxic activity is attributed to the contribution of the core of the complexes based on gold cyanide. From the results obtained, KAu(CN)_2_ displayed an IC_50_ value (0.06 µg/mL) well below (one order of magnitude) than that of the camphorimine gold complexes.

### 2.5. Redox Properties

**Figure 4:**
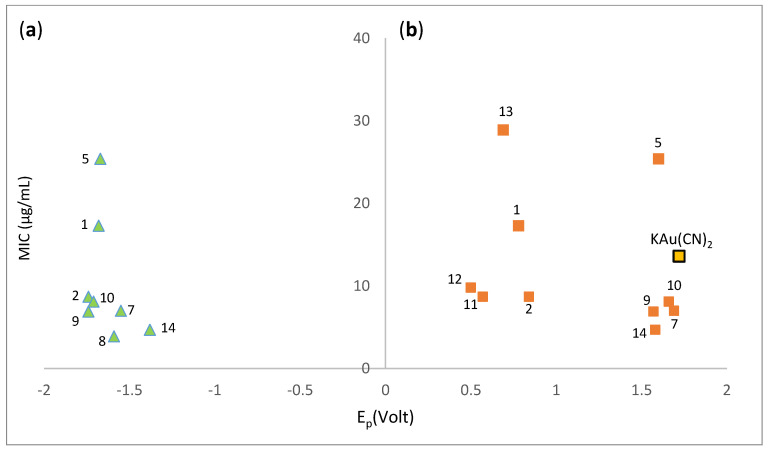
Relationship between the MIC values for *P. aeruginosa* 477 and the cathodic (**a**) and anodic (**b**) redox potentials for the complexes.

Electron transfer is often involved in biological processes, so the study of the electrochemical properties of the camphorimine gold complexes could allow some insight into their redox properties. The study was undertaken by cyclic voltammetry in acetonitrile using Bu_4_NBF_4_ as electrolyte (see Experimental for details). The data obtained is depicted in Table 1. As a general trend, the complexes display irreversible anodic waves at potentials lower than that of K[Au(CN)_2_] ($E_{p}^{ox}$ = 1.72 V, Figure 4a). The cathodic processes are also irreversible and fall within the potentials −1.65 and −1.85 V (Table 1). No cathodic process or adsorption wave indicative of gold formation were observed for K[Au(CN)_2_].

**Figure 5:**
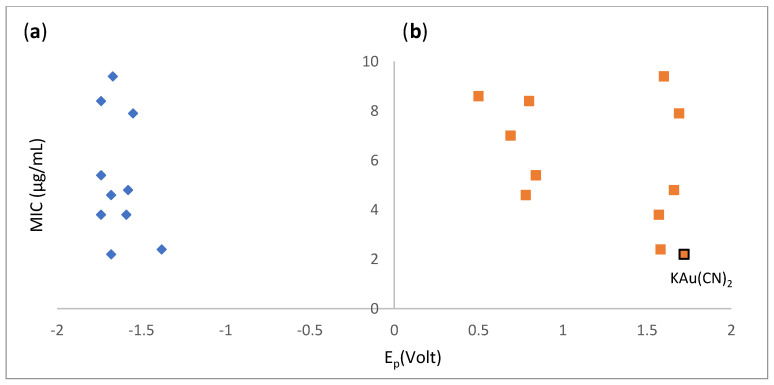
Relationship between the MIC values for *B. contaminans* and the redox potentials for the complexes.

The plots of the anodic and cathodic potentials versus the MIC values for *P. aeruginosa* 477 and *B. contaminans* were depicted in Figure 4 and Figure 5, respectively. A random distribution is observed in what refers to points defined by the MIC values and the reduction potentials (Figure 4a and Figure 5a) that fall in the range of values reported for the free ligands [5,26,27].

The anodic processes accommodate within two ranges of potentials either for *P. aeruginosa* or *B. contaminans* (Figure 4b and Figure 5b). The complexes with just one ligand (**1**,**2**,**11**,**13**) tend to fit into the range of the lower potentials, while those with two ligands (**5**,**7**,**10**) fit the potential values that include the KAu(CN)_2_ precursor. The electron releasing/withdrawing properties of the camphorimine ligands (L = OC_10_H_14_NY) may drive the redox and structure of the complexes, since electron donor ligands (Y = C_6_H_4_NH_2_, C_6_H_4_N; Z = *m*-C_6_H_4_, Scheme 1) tend to produce mono ligand-complexes that oxidize at the lower range of potentials.

## 3. Materials and Methods

The complexes were synthesized under nitrogen using Schlenk and vacuum techniques. Camphor ligands (OC_10_H_14_NY: Y = NH_2_, C_6_H_5_, C_6_H_4_NH_2_-4, C_6_H_4_CH_3_-4, C_6_H_4_OH-3, NC_10_H_14_NC_6_H_4_) were prepared according to reported procedures [27]. Gold potassium dicyanide, camphor, the amines, and hydrazine were purchased from Sigma Aldrich. Acetonitrile (PA grade) was purchased from Carlo Erba, purified by conventional techniques [28] and distilled before use. The FTIR spectra were obtained from KBr pellets using a JASCO FT/IR 4100 spectrometer. The NMR spectra (^1^H, ^13^C, DEPT, HSQC, and HMBC) were obtained from CD_3_CN, DMSO, or CDCl_3_ solutions using a Bruker Avance II+ (300 or 400 MHz) spectrometers. The NMR chemical shifts are referred to TMS (δ = 0 ppm). The redox properties were studied by cyclic voltammetry using a three compartments cell equipped with a Pt wire electrode and interfaced with a VoltaLab PST050 equipment. The cyclic voltammograms were obtained using solutions of NBu_4_BF_4_ in CH_3_CN (0.10 M) as electrolyte. The potentials were measured in Volts (±10 mV) versus SCE at 200 mV/s using [Fe(η^5^-C_5_H_5_)_2_]^0/+^ ($E_{1/2}^{red}$ = 0.382 V; CH_3_CN) as internal reference. The window of potential was established using a solution of the electrolyte [Bu_4_N][BF_4_] (0.10 M) in the absence of complex (Supplementary Materials).

### 3.1. Synthesis

K[Au(CN)_2_(OC_10_H_14_NC_6_H_4_NH_2_)]·H_2_O (**1**)—KAu(CN)_2_ (30 mg, 0.104 mmol) and OC_10_H_14_NC_6_H_4_NH_2_ (^A1^L, 27 mg, 0.104 mmol) were stirred under vacuum for ½ h. Acetonitrile (7 mL) was added and the mixture was stirred overnight at 25 °C. Slow evaporation of the solvent close to dryness allowed the red complex to precipitate. The residual solvent was decanted off and the compound dried under vacuum. Yield 40 mg, 69%; FTIR (KBr, cm^−1^): 3455, 3340, 2142, 1733, 1625, 1595, 428. Elem. Anal. (%) for KAuC_18_N_4_H_20_O·H_2_O, Found: C, 38.1; N, 10.2; H, 3.5. Calc.: C, 38.4; N, 10.0; H, 3.9. ^1^H NMR (CD_3_CN, 400 MHz, δ_ppm_): 6.87 (d, *J* = 8.6 Hz, 2H), 6.67 (d, *J* = 8.6 Hz, 2H), 4.32 (s, 2H), 2.97 (d, *J* = 4.8 Hz, 1H) 2.17–2.11 (m, 1H), 1.91–1.85 (m, 1H), 1.67–1.50 (m, 2H), 1.01 (s, 3H), 0.98 (s, 3H), 0.79 (s, 3H). ^13^C NMR (CD_3_CN, 400 MHz, δ_ppm_): 208.2, 169.1, 150.7, 148.1, 139.4, 125.1, 115.4, 58.5, 51.5, 45.7, 31.2, 24.6, 20.9, 17.8, 9.4.

[Au(CN)(OC_10_H_14_NC_6_H_4_NH_2_)]·CH_3_CN (**2**)—KAu(CN)_2_ (50 mg, 0.17 mmol) and OC_10_H_14_NC_6_H_4_NH_2_ (^A1^L, 33.3 mg, 0.13 mmol) were stirred under vacuum for ½ h. Acetonitrile (7 mL) was added and the mixture was stirred overnight at 25 °C. Upon slight evaporation, the gold suspension was separated by filtration. Full solvent evaporation led to precipitation of the complex as a light-brown precipitate. Yield 44 mg, 65%; FTIR (KBr, cm^−1^): 3455, 3340, 2142, 1733, 1625, 1594, 419. Elem. Anal. (%) for AuC_17_H_20_N_3_O·CH_3_CN, Found: C, 43.8; N, 10.4; H, 4.3. Calc.: C, 43.9; N, 10.8; H, 4.5. ^1^H NMR (CD_3_CN, 400 MHz, δ_ppm_): 6.87 (d, *J* = 8.7 Hz, 2H), 6.67 (d, *J* = 8.7 Hz, 2H), 4.35 (sbr, 2H), 2.98 (d, *J* = 4.8 Hz, 1H), 2.26–2.10 (m, 1H), 1.90–1.85 (m, 1H), 1.68–1.51 (m, 2H), 1.01 (s, 3H), 0.98 (s, 3H), 0.78 (s, 3H). ^13^C NMR (CD_3_CN, 400 MHz, δ_ppm_): 208.1, 169.1, 150.7, 148.1, 139.4, 125.1, 115.4, 58.4, 51.5, 45.7, 31.2, 24.6, 20.9, 17.8, 9.4.

[Au(CN)(OC_10_H_14_NC_6_H_4_NH_2_)_3_]·H_2_O (**3**)—KAu(CN)_2_ (50 mg, 0.17 mmol) and OC_10_H_14_NC_6_H_4_NH_2_ (^A1^L, 33 mg, 0.13 mmol) were stirred under vacuum for ½ h. Tetrahydrofuran (7 mL) was added to the mixture and the red solution stirred overnight under room temperature. The gold residues were filtered off and the solution evaporated. The oil obtained was washed with diethyl ether giving the light brown complex. Yield 23 mg, 52%. FTIR (KBr, cm^−1^): 3444, 3384, 2143, 1735, 1627, 1591, 409. Elem. Anal. (%) for AuC_49_H_62_N_7_O_4_·H_2_O, Found: C, 58.3; N, 9.7; H, 6.2. Calc.: C, 58.4; N, 9.7; H, 6.0. ^1^H NMR (CD_3_CN, 400 MHz, δ_ppm_): 6.87 (d, *J* = 8.6 Hz, 2H), 6.67 (d, *J* = 8.6 Hz, 2H), 4.32 (sbr, 2H), 2.97 (d, *J* = 4.7 Hz, 1H), 2.20–2.08 (m, 1H), 1.90–1.76 (m, 1H), 1.67–1.50 (m, 2H), 1.01 (s, 3H), 0.98 (s, 3H), 0.78 (s, 3H). ^13^C NMR (CD_3_CN, 400 MHz, δ_ppm_): 208.0, 169.1, 150.7, 148.0, 139.4, 125.1, 115.4, 58.4, 51.5, 45.7, 31.2, 24.6, 20.9, 17.8, 9.4.

K[Au(CN)_2_(OC_10_H_14_NC_6_H_4_CH_3_)] (**4**)—KAu(CN)_2_ (50 mg, 0.17 mmol) and OC_10_H_14_NC_6_H_4_CH_3_ (^A2^L, 43 mg, 0.17 mmol) were stirred under vacuum for ½ h. Acetonitrile (10 mL) was added forming a yellow solution. The mixture was stirred under reflux for 24 h. After cooling, the solvent was evaporated, forming an oil that was washed with ether affording the yellow complex that was filtered off the solution. Yield 46 mg, 50%. FTIR (KBr, cm^−1^): 3442, 2143, 1748, 1652, 426. Elem. Anal. (%) for KAuC_19_H_21_N_3_O, Found: C, 41.6; N, 7.6; H, 3.8. Calc.: C, 42.0; N, 7.7; H, 3.9. ^1^H NMR (CD_3_CN, 400 MHz, δ_ppm_): 7.22 (d, *J* = 7.5 Hz, 2H), 6.83 (d, *J* = 7.3 Hz, 2H), 2.80 (d, *J* = 3.6 Hz, 1H), 2.33 (s, 3H), 2.15–2.02 (m, 1H), 1.91–1.84 (m, 1H), 1.66–1.53 (m, 2H), 1.03 (s, 3H), 0.97 (s, 3H), 0.84 (s, 3H). ^13^C NMR (CD_3_CN, 400 MHz, δ_ppm_): 207.7, 172.8, 150.7, 148.0, 136.2, 130.5, 121.4, 58.8, 51.0, 45.3, 30.9, 24.8, 21.1, 20.9, 17.6, 9.3.

[Au(CN)(OC_10_H_14_NC_6_H_4_CH_3_)_2_]·½CH_3_CN (**5**)—KAu(CN)_2_ (30 mg, 0.104 mmol) and OC_10_H_14_NC_6_H_4_CH_3_ (^A2^L, 51 mg, 0.20 mmol) were stirred under vacuum for ½ h. Then, acetonitrile (7 mL) was added, and the mixture stirred for 3 days. Upon solvent evaporation, a yellow precipitate was obtained that was washed with ether and dried under vacuum. Yield 57 mg, 73%. FTIR (KBr, cm^−1^): 3446, 2153, 2142, 1747, 1652, 427. Elem. Anal. (%) for AuC_35_H_42_N_3_O_2_·½CH_3_CN, Found: C, 57.1; N, 6.7; H, 5.7. Calc.: C, 57.3; N, 6.5; H, 5.8. ^1^H NMR (CDCl_3_, 400 MHz, δ ppm): 7.18 (d, *J* = 7.8 Hz, 2H), 6.86 (d, *J* = 7.9 Hz, 2H), 2.86 (d, *J* = 4.7 Hz, 1H), 2.36 (s, 3H), 2.13–2.05 (m, 1H), 1.91–1.81 (m, 1H), 1.73–1.59 (m, 2H), 1.11 (s, 3H), 0.98 (s, 3H), 0.89 (s, 3H). ^13^C NMR (CDCl_3_, 400 MHz, δ ppm): 206.8, 171.5, 147.0, 135.3, 129.7, 120.9, 58.2, 50.3, 44.8, 30.3, 24.5, 21.1, 21.0, 17.7, 9.2

[{Au(CN)}_2_(OC_10_H_14_NC_6_H_4_CH_3_)]·2H_2_O (**6**)—KAu(CN)_2_ (30 mg, 0.104 mmol) and OC_10_H_14_NC_6_H_4_CH_3_ (^A2^L, 20 mg, 0.080 mmol) were stirred under vacuum for ½ h. Then, acetonitrile (7 mL) was added, and the mixture stirred overnight at 25 °C. Upon slight concentration, the unreacted reagent was separated by filtration. The light-yellow complex was obtained upon solvent removal by evaporation. Yield 35.4 mg, 60%. FTIR (KBr, cm^−1^): 3435, 2142, 1747, 1651, 429. Elem. Anal. for Au_2_C_19_H_21_N_3_O·2H_2_O Found: C, 30.8; N, 5.8; H, 3.0. Calc.: C, 31.0; N, 5.7; H, 3.4. ^1^H NMR (CD_3_CN, 300 MHz δ_ppm_): 7.22 (d, *J* = 8.0 Hz, 2H), 6.83 (d, *J* = 8.0 Hz, 2H), 2.79 (d, *J* = 4.7 Hz, 1H), 2.33 (s, 3H), 2.16–2.06 (m, 1H), 1.90–1.84 (m, 1H), 1.66–1.53 (m, 2H) 1.03 (s, 3H), 0.97 (s, 3H), 0.83 (s,3H). ^13^C NMR (CD_3_CN, 300 MHz δ_ppm_): 207.7, 172.7, 150.7, 148.0, 136.1, 130.6, 121.4, 58.8, 51.0, 45.2, 30.9, 24.8, 21.1, 20.9, 17.6, 9.4.

K[Au(CN)_2_(OC_10_H_14_NC_6_H_4_CH_3_)_2_]·^1^/_2_Et_2_O (**7**)—KAu(CN)_2_ (50 mg, 0.17 mmol) and OC_10_H_14_NC_6_H_4_CH_3_ (^A2^L, 43 mg, 0.17 mmol) were stirred under vacuum for ½ h. Acetonitrile (10 mL) was added and the yellow solution was stirred under reflux for 24 h. After cooling, the residual gold was filtered off and the solvent was evaporated forming an oil that was washed with ether affording the yellow complex. Yield 48.7 mg, 68%. FTIR (KBr, cm^−1^): 2155, 2142, 1747, 1653, 428. Elem. Anal. (%) for KAuC_36_H_42_N_4_O_2_·^1^/_2_Et_2_O, Found: C, 54.9; N, 6.8; H, 5.3. Calc.: C, 54.6; N, 6.7; H, 5.7. ^1^H NMR (DMSO, 400 MHz, δ_ppm_): 7.21 (d, *J* = 8.0 Hz, 2H), 6.82 (d, *J* = 8.1 Hz, 2H), 2.74 (d, *J* = 4.6 Hz, 1H), 2.30 (s, 3H), 2.09–2.00 (m, 1H), 1.90–1.80 (m, 1H), 1.61–1.48 (m, 2H), 0.98 (s, 3H), 0.93 (s, 3H), 0.78 (s, 3H). ^13^C NMR (DMSO, 400 MHz, δ_ppm_): 205.8, 171.2, 149.6, 146.7, 134.5, 129.6, 120.2, 57.5, 49.6, 44.1, 29.5, 23.6, 20.5, 20.4, 17.1, 9.0.

K_3_[{Au(CN)_2_}_3_(OC_10_H_14_NC_6_H_4_OH-*m*)] (**8**)—KAu(CN)_2_ (50 mg, 0.17 mmol) and OC_10_H_14_NC_6_H_4_OH-*m* (^A4^L, 88 mg, 0.26 mmol) were stirred under vacuum for ½ h. Acetonitrile (7 mL) was added to the mixture and the yellow suspension was stirred overnight. Upon filtration to remove residues, the solution was evaporated, and the precipitate washed with diethyl ether affording a dark yellow complex. Yield 30 mg, 47%; FTIR (KBr, cm^−1^): 3421, 2141, 1749, 1656, 426. Elem. Anal. (%) for K_3_Au_3_C_22_H_19_N_7_O_2_, Found: C, 23.1; N, 8.6; H, <2. Calc.: C, 23.6; N, 8.7; H, 1.7. ^1^H NMR (CD_3_CN, 400 MHz, δ_ppm_): 7.21 (t, *J* = 7.8 Hz, 1H), 6.63 (d, *J* = 8.0 Hz, 1H), 6.37 (d, *J* = 7.8 Hz, 1H), 6.34 (s, 1H), 2.77 (d, *J* = 4.1 Hz, 1H), 2.30 (s, 1H), 2.12–2.02 (m, 1H), 1.91–1.84 (m, 1H), 1.64–1.53 (m, 2H), 1.03 (s, 3H), 0.97 (s, 3H), 0.85 (s, 3H). ^13^C NMR (CD_3_CN, 400 MHz, δ_ppm_): 207.8, 173.2, 158.6, 152.4, 150.8, 131.1, 113.0, 112.3, 107.7, 58.9, 51.1, 45.1, 30.8, 24.8, 21.2, 17.6, 9.3.

K[Au(CN)_2_(OC_10_H_14_NNH_2_)] (**9**)—KAu(CN)_2_ (70 mg, 0.24 mmol) and OC_10_H_14_NC_6_H_4_CH_3_ (^A5^L, 43.8 mg, 0.24 mmol) were stirred under vacuum for ½ h. Then, acetonitrile (7 mL) was added, and the mixture stirred overnight. The solvent was evaporated until precipitation started and the suspension was kept in the fridge overnight. A light-yellow precipitate was obtained that was filtered off solution and dried under vacuum. A further crop of the complex was carried out upon concentration and kept in the fridge for a few days. Yield 61 mg, 54%. FTIR (KBr, cm^−1^): 3414, 3295, 2154, 2142, 1706, 1575, 427. Elem. Anal. (%) for KAuC_12_H_16_N_4_O Found: C, 30.5; N, 11.8; H, 3.3; Calc.: C, 30.8; N, 12.0; H, 3.4. ^1^H NMR (DMSO, 400 MHz, δ_ppm_): 7.43 (s, 2H); 2.98 (d, *J* = 3.9 Hz), 1,93–1.85 (m, 1H), 1.74-1.64 (m, 1H), 1.33–1.21 (m, 2H), 0.91 (s, 3H), 0.85 (s, 3H), 0.73 (s, 3H). ^13^C NMR (DMSO, 400 MHz, δ_ppm_): 203.6, 149.5, 145.8, 57.3, 44.9, 44.5, 30.9, 23.4, 19.9, 17.9, 9.1.

K[Au(CN)_2_(OC_10_H_14_NOH)_2_]·^1^/_2_H_2_O (**10**)—KAu(CN)_2_ (50 mg, 0.17 mmol) and C_10_H_14_NC_6_H_4_N (^A6^L, 60 mg, 0.33 mmol) were stirred under vacuum for ½ h. Acetonitrile (7 mL) was added and the mixture stirred overnight. The light-yellow complex was obtained upon evaporation of the solvent. Yield 98 mg, 87%. FTIR (KBr, cm^−1^): 3442, 2144, 1743, 1739, 1642, 425. Elem. Anal. (%) for KAuC_22_H_30_N_4_O_4_·^1^/_2_H_2_O, Found: C, 40.0; N, 8.5; H, 4.5. Calc.: C, 40.0; N, 8.5; H, 4.7. ^1^H NMR (DMSO, 300 MHz δ_ppm_): 11.94 (s, 1H), 3.11 (d, *J* = 4.1 Hz, 1H), 2.03–1.92 (m, 1H), 1.82–1.72 (m, 1H), 1.45–1.27 (m, 2H), 0.93 (s, 3H), 0.90 (s, 3H), 0.75 (s, 3H). ^13^C NMR (DMSO, 300 MHz δ_ppm_): 203.8, 158.6, 149.8, 57.9, 46.0, 44.4, 30.1, 23.5, 20.2, 17.4, 9.0.

K_3_[{Au(CN)_2_}_3_(C_10_H_14_NC_6_H_4_N)]·H_2_O (**11**)—KAu(CN)_2_ (50 mg, 0.17 mmol) and C_10_H_14_NC_6_H_4_N (^C^L, 42 mg, 0.17 mmol) were stirred under vacuum for ½ h. Acetonitrile (10 mL) was added and the orange solution stirred overnight. The solvent was evaporated until precipitation started and the gold residues were separated by filtration. The brown complex was obtained by evaporation of the solution. Yield 27 mg, 43%. FTIR (KBr, cm^−1^): 3433, 2142, 1631, 759, 427. Elem. Anal. (%) for K_3_Au_3_C_22_H_18_N_8_, Found: C, 23.3; N, 10.1; H, <2. Calc.: C, 23.6; N, 10.0; H, 1.8. ^1^H NMR (CD_3_CN, 400 MHz, δ_ppm_): 7.96–7.94 (m, 2H), 7.67–7.64 (m, 2H), 3.00 (d, *J* = 4.4 Hz, 1H), 2.25–2.38 (m, 1H), 2.13–2.04 (m, 1H), 1.37 (s, 3H), 1.36–1.30 (m, 2H), 1.11 (s, 3H), 0.58 (s, 3H). ^13^C NMR (CD_3_CN, 400 MHz, δ_ppm_): 166.6, 164.9, 150.6, 142.4, 142.3, 129.6, 129.5, 129.0, 128.9, 54.9, 54.5, 54.0, 32.5, 25.2, 20.4, 18.7, 10.3.

K[Au(CN)_2_(C_10_H_14_NC_6_H_4_N)_3_]·H_2_O (**12**)—KAu(CN)_2_ (50 mg, 0.17 mmol) and C_10_H_14_NC_6_H_4_N (^C^L, 81 mg, 0.34 mmol) were stirred under vacuum for ½ h. Acetonitrile (7 mL) was added to the mixture and the reaction stirred for 48 h. The solution was evaporated, and the unreacted gold compound was filtered off. The complex was obtained as a brown complex upon solvent evaporation under vacuum. Yield 72 mg, 62%; FTIR (KBr, cm^−1^): 3445, 2155, 2142, 1631, 756, 426. Elem. Anal. (%) for KAuC_49_H_51_N_8_·H_2_O, Found: C, 58.9; N, 11.4; H, 5.2. Calc.: C, 58.8; N, 11.0; H, 5.5. ^1^H NMR (CD_3_CN, 400 MHz, δ_ppm_): 7.97–7.92 (m, 2H), 7.67–7.63 (m, 2H), 3.00 (d, *J* = 4.2 Hz, 1H), 2.36–2.26 (m, 1H), 2.13–2.00 (m, 2H), 1.36 (s, 3H), 1.35–1.28 (m, 1H), 1.11 (s, 3H), 0.56 (s, 3H). ^13^C NMR (CD_3_CN, 400 MHz, δ_ppm_): 166.5, 164.9, 150.6, 142.4, 142.3, 129.6, 129.5, 129.0, 128.9, 54.9, 54.5, 54.0, 32.5, 25.2, 20.4, 18.6, 10.3.

[Au(CN){(OC_10_H_14_N)_2_C_6_H_4_-*m*}]·CH_3_CN (**13**)—Upon addition of acetonitrile (7 mL) to the vacuum pumped mixture of KAu(CN)_2_ (30 mg, 0.104 mmol) and (OC_10_H_14_N)_2_C_6_H_4_-*m* (^B1^L, 31 mg; 0.077 mmol) a dark yellow suspension was obtained that was stirred overnight at RT. Filtration was carried out to remove unreacted gold reagent, followed by solvent evaporation to afford the yellow complex. Yield 37 mg, 72%. FTIR (KBr, cm^−1^): 3459, 2142, 1753, 1678, 428. Elem. Anal. for AuC_27_H_32_N_3_O_2_·CH_3_CN, Exp.: C, 52.4; N, 8.1; H, 5.2. Calc.: C, 52.1; N, 8.4; H, 5.3. ^1^H NMR (CD_3_CN, 400 MHz, δ_ppm_): 7.41 (t, *J* = 7.9 Hz, 1H), 6.71 (d, *J* = 7.9 Hz, 2H), 6.34 (s, 1H), 2.77 (d, *J* = 4.7 Hz, 2H) 2.06–2.04 (m, 1H), 1.91–1.86 (m, 2H), 1.64–1.55 (m, 5H), 1.04 (s, 6H), 0.97 (s, 6H), 0.87 (s, 6H). ^13^C NMR (CD_3_CN, 400 MHz, δ_ppm_): 207.5, 173.8, 152.0, 150.7, 130.9, 117.4, 111.4, 59.0, 51.1, 45.1, 30.9, 24.8, 21.2, 17.6, 9.4.

K_3_[{Au(CN)_2_}_3_{(OC_10_H_14_N)_2_C_6_H_4_-*p*}]·3H_2_O (**14**)—KAu(CN)_2_ (50 mg, 0.17 mmol) and (OC_10_H_14_N)_2_C_6_H_4_-*p* (^B2^L, 33 mg, 0.22 mmol) were stirred under vacuum for ½ h. The mixture was dissolved in acetonitrile (7 mL) and stirred overnight. The solution was concentrated and filtered to remove impurities. Evaporation produced a light-yellow complex. Yield 40 mg, 53%; FTIR (KBr, cm^−1^): 3434, 2142, 1755, 1685, 427. Elem. Anal. (%) for K_3_Au_3_C_32_H_32_N_8_O_2_·3H_2_O, Found: C, 28.8; N, 8.7; H, 2.2. Calc.: C, 29.1; N, 8.5; H, 2.9. ^1^H NMR (CD_3_CN, 400 MHz, δ_ppm_): 6.99 (s, 4H), 2.87 (d, *J* = 4.7 Hz, 2H), 2.16–2.09 (m, 3H), 1.92–1.87 (m, 1H), 1.69–1.56 (m, 4H), 1.05 (s, 6H), 1.00 (s, 6H), 0.86 (s, 6H). ^13^C NMR (CD_3_CN, 400 MHz, δ_ppm_): 207.5, 173.2, 150.7, 148.0, 122.4, 58.9, 51.2, 45.3, 30.9, 24.8, 21.1, 17.6, 9.4.

### 3.2. Computational Calculations

The optimization of the structures and the molecular geometry of the complexes were carried out by DFT calculations using GAMESS-US [22] version R3, with a B3LYP functional, using a SBKJC basis set [29]. The structures were confirmed stationary points by Hessians with non-negative eigen values and six near zero rotational and translational frequencies.

### 3.3. Biological Assays

#### 3.3.1. Bacterial Strains

The bacterial strains *E. coli* ATCC25922, *P. aeruginosa* 477, *B. contaminans* IST408, and *S. aureus* Newman were used in the present work and were kept at −80 °C in 20% glycerol. When in use, bacterial strains were maintained in Luria Broth (LB) solid media (Sigma).

#### 3.3.2. Minimal Inhibitory Concentration Assessment

The Au(I) complexes minimal inhibitory concentration (MIC) towards the above-mentioned bacterial strains was determined by microdilution methodologies using Mueller–Hinton broth (MH), as previously described [30]. Positive (no compound) and negative controls (no bacterial inoculum) were included in each microdilution experiment.

#### 3.3.3. Toxicity Assessment in Normal Cells

The in vitro cytotoxicity of the gold complexes was evaluated in adult human dermal fibroblasts (HDF) (Sigma-Aldrich) using the MTT assay as previously described [29]. Cells were cultured in Fibroblast Growth Medium (Sigma-Aldrich) following the instructions of the supplier for these cells. Cells laid in 96 well plates were incubated with serial dilutions of the compounds for 24 h. Each compound’s dilution was performed in four wells. For each assay controls (cells without test compound) were done. At least two independent experiments were performed for each cytotoxicity analysis. The cytotoxicity of each compound was expressed by the IC_50_, the concentration of compound causing 50% decrease of cellular viability.

## 4. Conclusions

Camphorimine gold(I) cyanide complexes display relevant antibacterial activity towards Gram-negative *E. coli* ATCC25922 and Gram-positive *S. aureus* Newman bacterial strains and a remarkably high antibacterial activity towards the Gram-negative strains *P. aeruginosa* 477 and *B. contaminans* IST408. Depending on the characteristics of the camphorimine ligands, the gold content and the structural arrangement, the complexes reach rather low MIC values (2.4 µg/mL for *B. contaminans* IST408 (**14**) or 3.9 µg/mL for *P. aeruginosa* 477 (**8**), revealing excellent antibacterial properties. According to DFT calculations, release of KCN by KAu(CN)_2_ is favored by interaction with the camphorimine ligands with formation of species [Au(CN)L] that may add another gold unit [{Au(CN)}_2_L] or ligand [Au(CN)L_m_].

An insight into the relationship between the MIC values and the gold content shows that an inverse correlation exists, i.e., the higher the Au content, the lower the MIC values. The plots of the MIC values versus Au (%) reveal that the performance of the complexes towards the two strains is distinct. In the case of *B. contaminans*, the antimicrobial activity correlates directly with that of the potassium gold cyanide precursor, while in the case of *P. aeruginosa*, it is essentially independent of it. Except for compound **14**, complexes of general formula K[Au(CN)_2_L] (**1**,**4**,**9**) and K[Au(CN)_2_L_2_] (**10**) display the highest activities against *B. contaminans*. These complexes have in common camphorimine ligands with amine (^A1^L,^A5^L) and hydroxy (^A6^L) substituents, which are easily ionizable, a fact that is considered to improve their low MIC values supported by the observation that the highest MIC value was found for complex [Au(CN)L_2_] (**5**) with low ionizable character.

In the case of *P. aeruginosa*, the plots show that there is no correlation between the values (MIC and % Au) for the Au(I) camphorimine complexes and the precursor KAu(CN)_2_, which sits aside from all lines. In this case, the MIC values for most of the complexes are lower than that of KAu(CN)_2_ and there is no evidence for enhancement of the antibacterial activity enabled by protic or hydrogen bonding substituents at the camphorimine ligand. Such trend shows that the coordination of the camphorimine ligands have a positive effect on the antibacterial activity, independently of the substituent.

No correlation was found between the redox potentials and the MIC values, in agreement with structural rather than electronic aspects are driving the interaction of the complexes with bacteria cells.

## Figures and Tables

**Table 1:** Biological and cyclic voltammetry data ^a^ for Au(I) complexes.

				E (Volt)		MIC (µg/mL)			
**Compound**		**Y** or **Z**	% Au	$E_{p}^{ox}$	$E_{p}^{red}$	*E. coli* ATCC25922	*P. aeruginosa* 477	*B. contaminans* IST408	*S. aureus* Newman
K[Au(CN)_2_(^A1^**L**)]·H_2_O	**1**	C_6_H_4_NH_2_ ^A1^**L**	35.0	0.78	−1.68	29.7 ± 1.9	17.3 ± 2.1	4.6 ± 0.5	32.7 ± 4.7
[Au(CN)(^A1^**L**)]·CH_3_CN	**2**		39.2	0.84	−1.74	49.8 ± 3.7	7.9 ± 1.8	4.5 ± 0.4	29.7 ± 1.1
[Au(CN)(^A1^**L**)_3_]·H_2_O	**3**		19.9	0.80	−1.74	250	9.1 ± 1.9	8.4 ± 1.2	151.5 ± 10.8
K[Au(CN)_2_(^A2^**L**)]	**4**	C_6_H_4_CH_3_ ^A2^**L**	36.2			44.3 ± 6.1	5.6 ± 0.1	4.2 ± 0.1	>250
[Au(CN)(^A2^**L**)_2_]·½CH_3_CN	**5**		26.8	1.6	−1.67	87.4 ± 6.4	28.1 ± 8.3	9.4 ± 1.4	250
[{Au(CN)}_2_(^A2^**L**)]·2H_2_O	**6**		53.4			46 ± 3.7	17.3 ± 1.3	5.3 ± 0.3	44.5 ± 4.3
K[Au(CN)_2_(^A2^**L**)_2_]	**7**		24.3	1.69	−1.55	34.03 ± 1.6	7.0 ± 0.8	7.9 ± 0.5	>250
K_3_[{Au(CN)_2_}_3_(^A4^**L**)]	**8**	*m*-OHC_6_H_4_^A4^**L**	52.7		−1.59	49.7 ± 1.2	3.9 ± 0.5	3.8 ± 0.2	30.8 ± 6.4
K[Au(CN)_2_(^A5^**L**)]	**9**	NH_2_ ^A5^**L**	42.1	1.57	−1.74	56.1 ± 6.8	6.9 ± 0.3	3.8 ± 0.2	53.9 ± 2.8
K[Au(CN)_2_(^A6^**L**)_2_] ·½H_2_O	**10**	OH ^A6^**L**	30.3	1.66	−1.58	17.3 ± 0.3	8.1 ± 0.6	4.8 ± 0.3	32.9 ± 1.6
K_3_[{Au(CN)_2_}_3_(^C^**L**)]·H_2_O	**11**	NC_6_H_4_N ^C^**L**	53.6			47.5 ± 6.8	8.7 ± 1.4	5.4 ± 0.4	47.1 ± 1.2
K[Au(CN)_2_(^C^**L**)_3_]·H_2_O	**12**		19.6	0.50	–	114.3 ± 11.9	9.8 ± 0.3	8.6 ± 0.6	>250
[Au(CN)(^B1^**L**)]·CH_3_CN	**13**	*m*-C_6_H_4_ ^B1^**L**	29.5	0.69	–	56.5 ± 1.2	28.9 ± 2.1	7.0 ± 0.2	81.2 ± 5.2
K_3_[{Au(CN)_2_}_3_(^B2^**L**)]· 3H_2_O	**14**	*p*-C_6_H_4_ ^B2^**L**	47.5	1.58	−1.38	23.5 ± 0.7	4.7 ± 0.4	2.4 ± 0.1	43.2 ± 1.7
KAu(CN)_2_			68.7	1.72	–	14.7 ± 0.1	13.6 ± 0.7	2.2 ± 0.02	15.6 ± 0.9

^a^ In Bu_4_NBF_4_/CH_3_CN (0.1 M) using a Pt wire working electrode. Potentials in Volt (±10 mV) measured versus SCE using Fe(C_5_H_5_)_2_]^0/+^ ($E_{1/2}^{ox}$ = 0.38 V) as internal reference.

## Data Availability

Not applicable.

## Supplementary Materials

FTIR and NMR (^1^H, ^13^C) spectra are available for all the new complexes (1–14) at https://www.mdpi.com/article/10.3390/antibiotics10101272/s1.
