# Supplementary material for: Synthesis and Characterization of Camphorimine Au(I) Complexes with a Remarkably High Antibacterial Activity towards B. contaminans and P. aeruginosa
Source: Antibiotics (Basel). 2021 Oct 19;10(10):1272. doi: 10.3390/antibiotics10101272 (PMC8532832; doi:10.3390/antibiotics10101272)

Synthesis and characterization of camphorimine Au(I) complexes with a remarkably high antibacterial activity towards *B. contaminans* and *P. aeruginosa*

Joana P. Costa [1], Sílvia A. Sousa [2,3], Catarina Soeiro [2,3], Jorge H. Leitão [2,3]*, Adelino M. Galvão [1], Fernanda Marques, [4] M. Fernanda N.N. Carvalho [1]*

1 Centro de Química Estrutural e Departamento de Engenharia Química, Instituto Superior Técnico, Universidade de Lisboa, Av. Rovisco Pais, 1049-001 Lisboa, Portugal

2 Instituto de Bioengenharia e Biociências, Departamento de Bioengenharia, Instituto Superior Técnico Universidade de Lisboa, Av. Rovisco Pais, 1049-001 Lisboa, Portugal.

3 Associate Laboratory, i4HB—Institute for Health and Bioeconomy at Instituto Superior Técnico, Universidade de Lisboa, Av. Rovisco Pais, 1049-001 Lisboa, Portugal

4 C[2]TN, Centro de Ciências e Tecnologias Nucleares Instituto Superior Técnico, Departamento de Engenharia e Ciências Nucleares, Universidade de Lisboa, Estrada Nacional 10 (km 139,7), 2695-066 Bobadela LRS, Portugal

* Correspondence: fcarvalho@tecnico.ulisboa.pt (M.F.N.N.C.); jorgeleitao@tecnico.ulisboa.pt (J.H.L.); Tel.: +351-218419178 (M.F.N.N.C.)

SUPPLEMENTARY DATA

Compound **1** - $K[Au(CN)_2(OC_{10}H_{14}NC_6H_4NH_2)]\cdot H_2O$

- FTIR

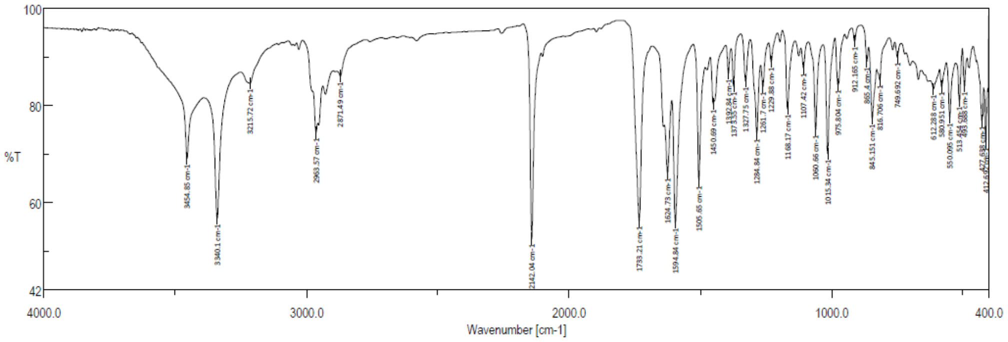

- ^{1}H NMR

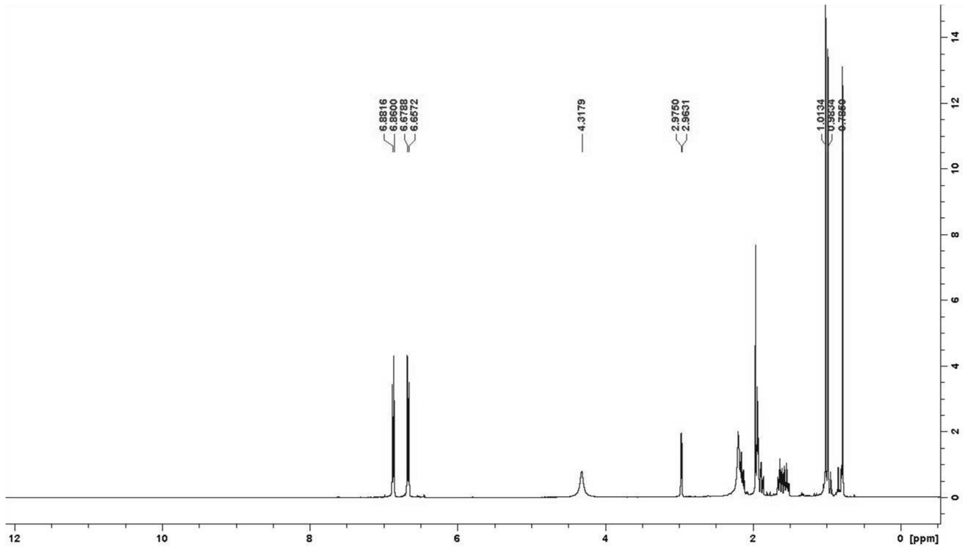

- ^{13}C NMR

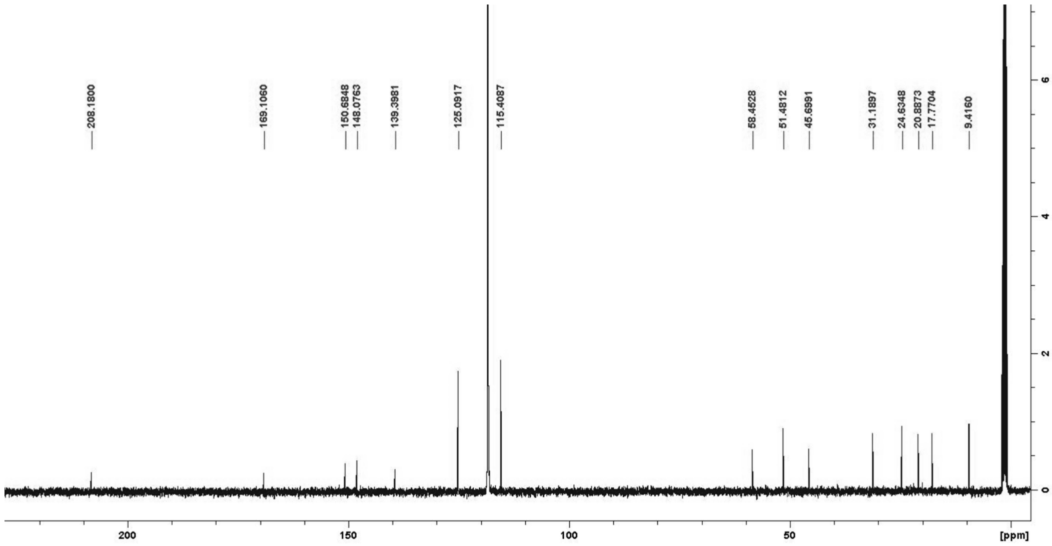

Compound **2** - [Au(CN)($OC_{10}H_{14}NC_6H_4NH_2$)]·CH_3CN

- FTIR

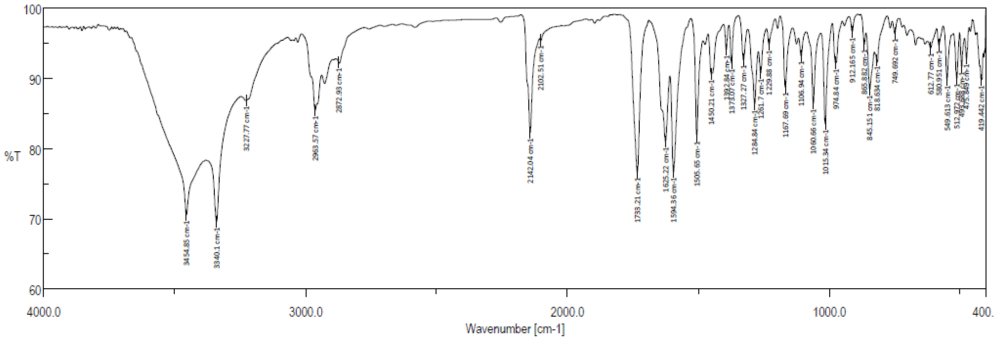

- ^{1}H NMR

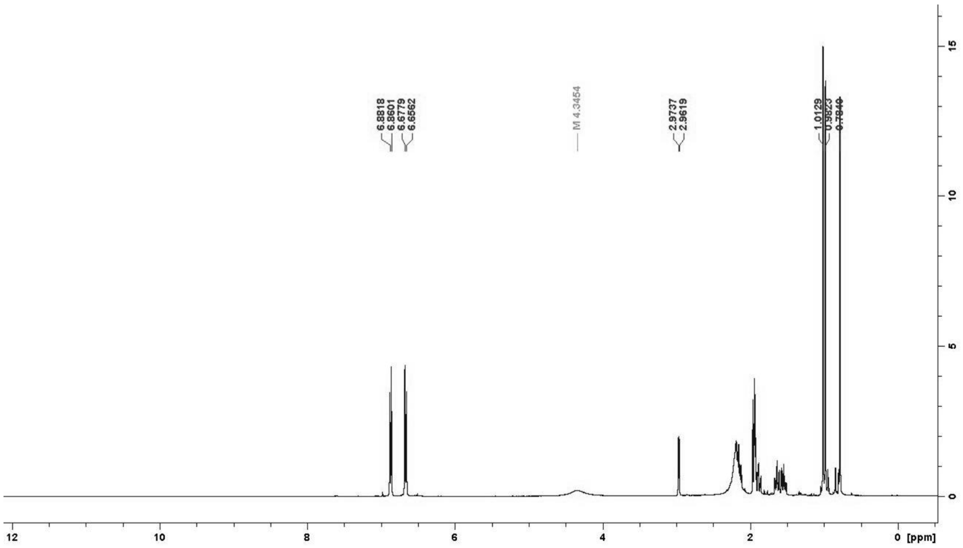

- ^{13}C NMR

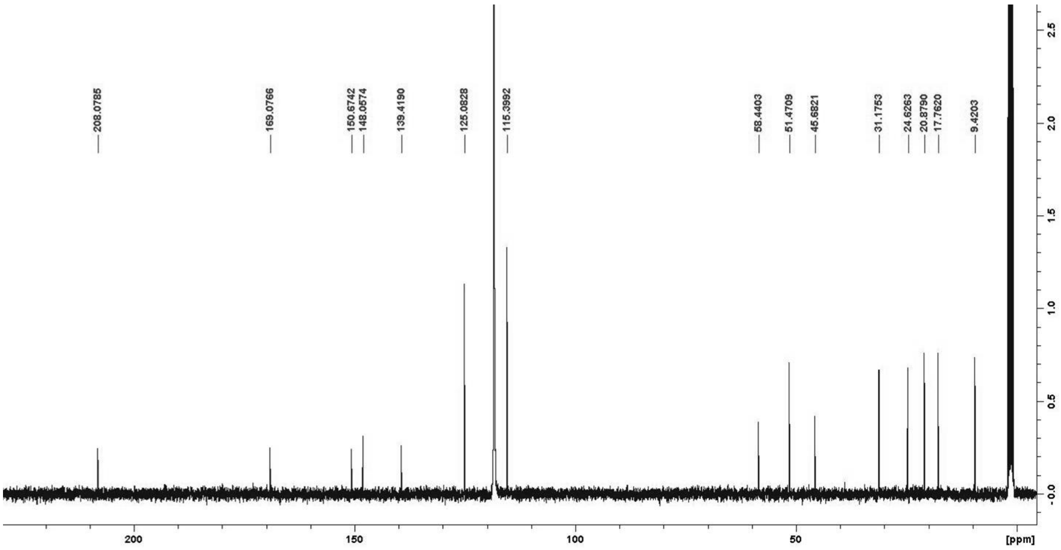

Compound **3** - $[Au(CN)(OC_{10}H_{14}NC_6H_4NH_2)_3]\cdot H_2O$

- FTIR

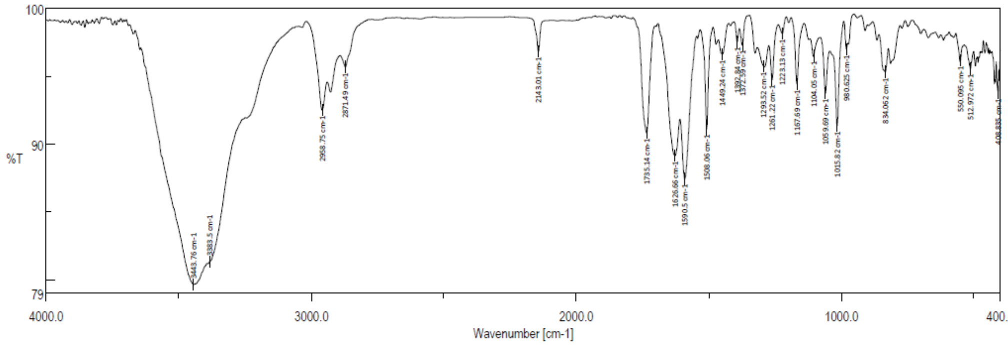

- ^1H NMR

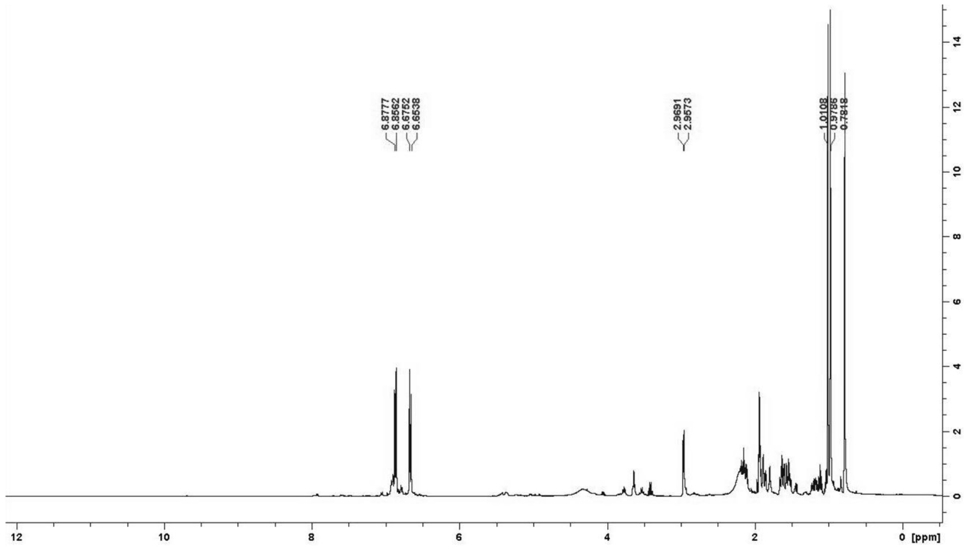

- ^{13}C NMR

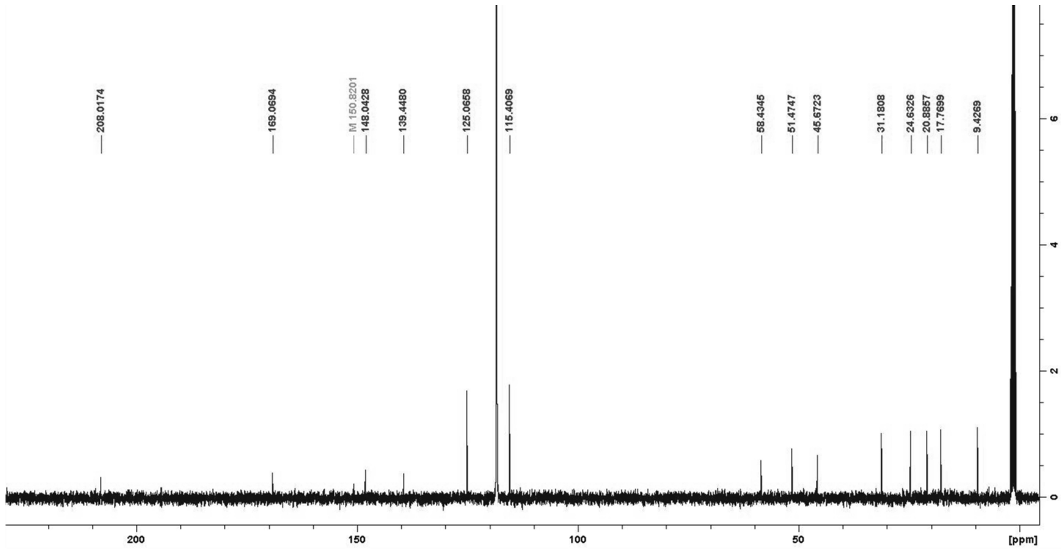

Compound **4** - $K[Au(CN)_2(OC_{10}H_{14}NC_6H_4CH_3)]$

- FTIR

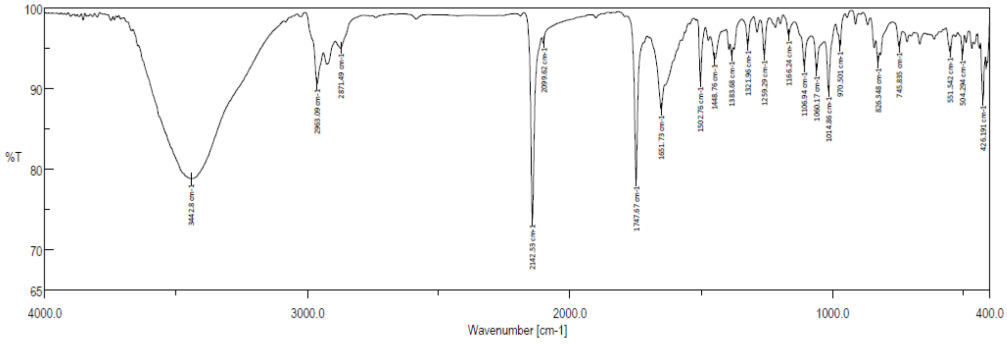

- ^{1}H NMR

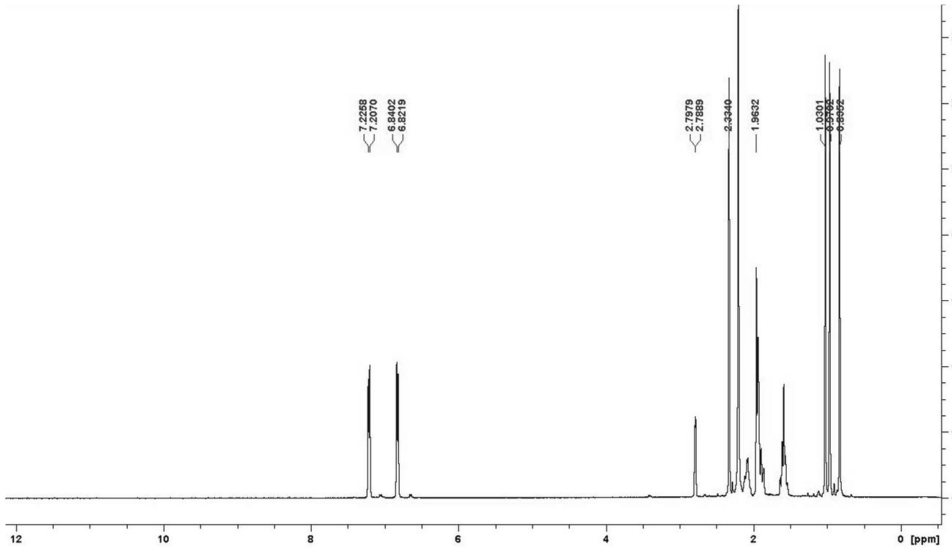

- ^{13}C NMR

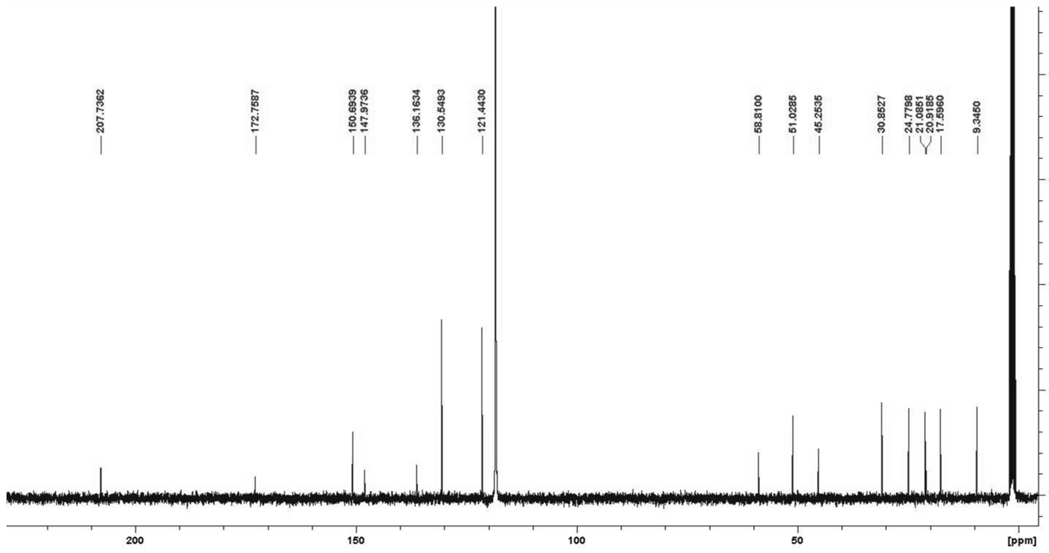

Compound **5** - $[Au(CN)(OC_{10}H_{14}NC_6H_4CH_3)_2]\cdot\frac{1}{2}CH_3CN$

- FTIR

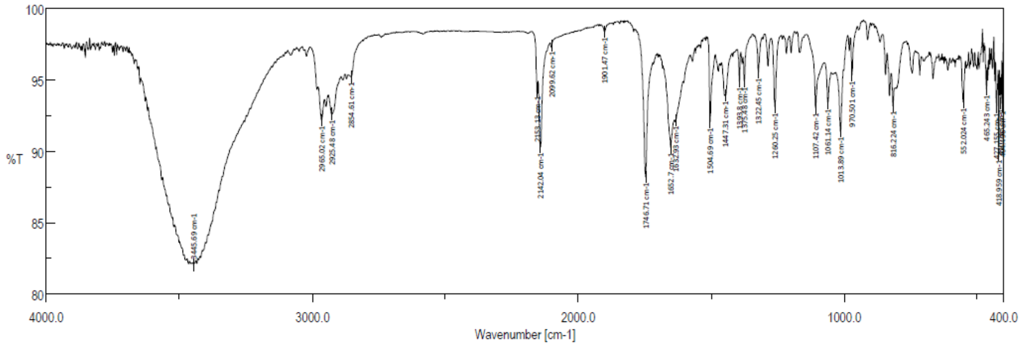

- ^1H NMR

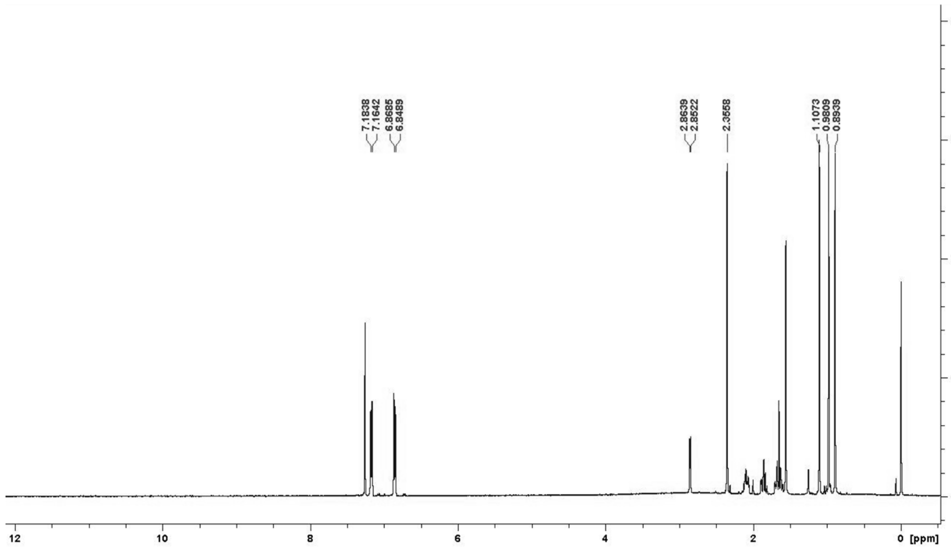

- ^{13}C NMR

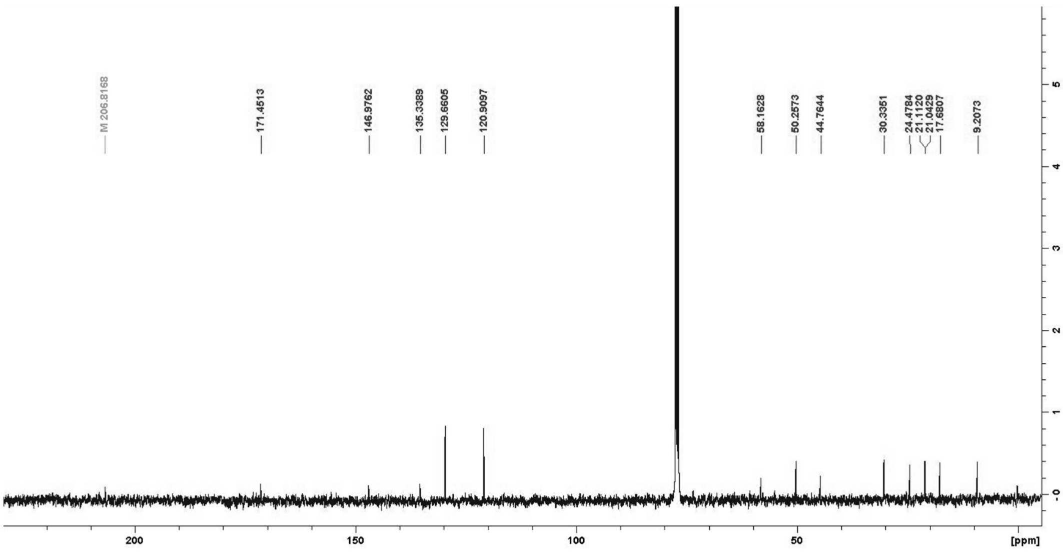

Compound **6** - $[\{Au(CN)\}_2(OC_{10}H_{14}NC_6H_4CH_3)]\cdot 2H_2O$

- FTIR

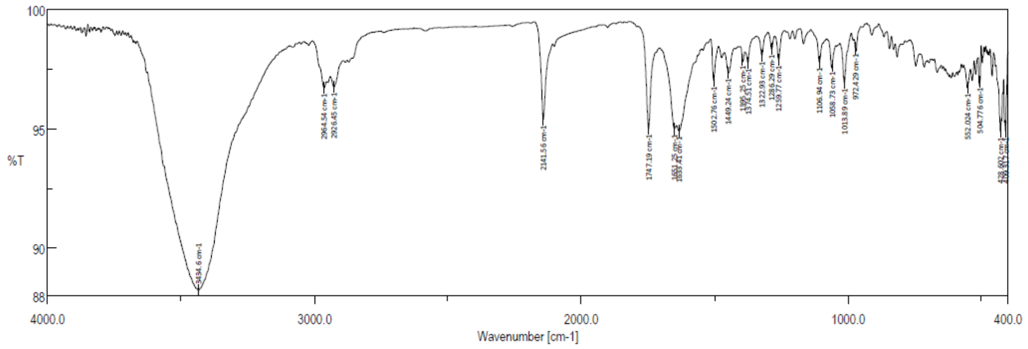

- ^{1}H NMR

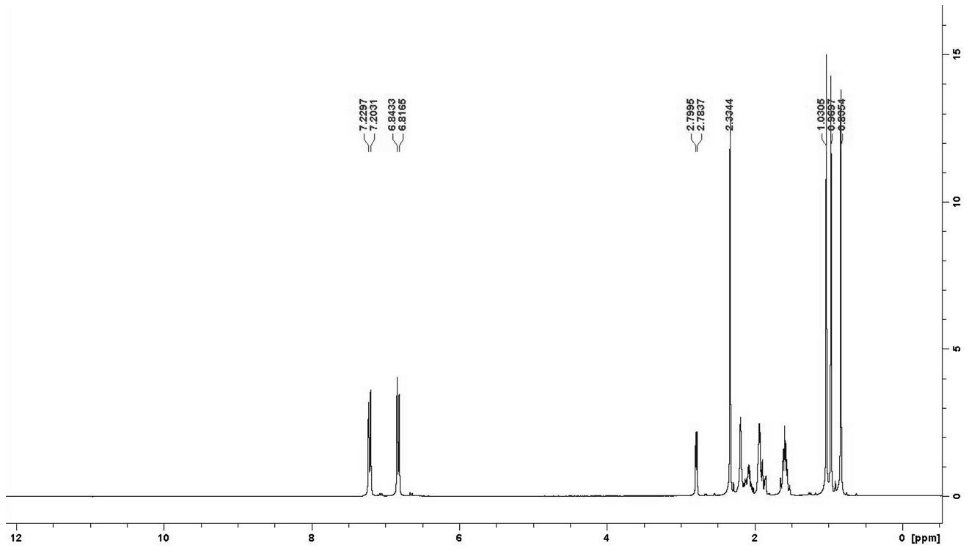

- ^{13}C NMR

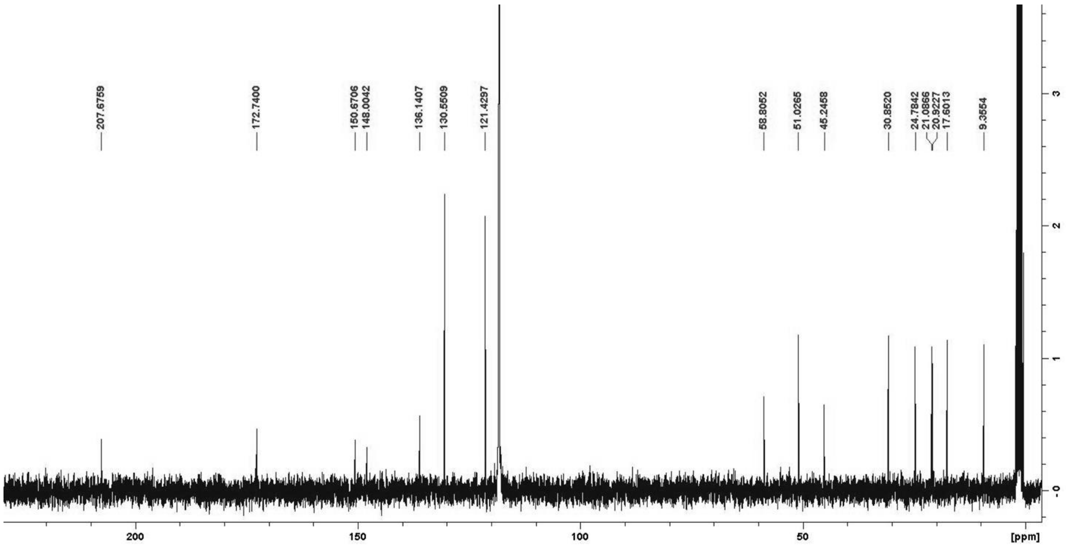

Compound **7** - $K[Au(CN)_2(OC_{10}H_{14}NC_6H_4CH_3)_2]\cdot{}^1/_2Et_2O$

- FTIR

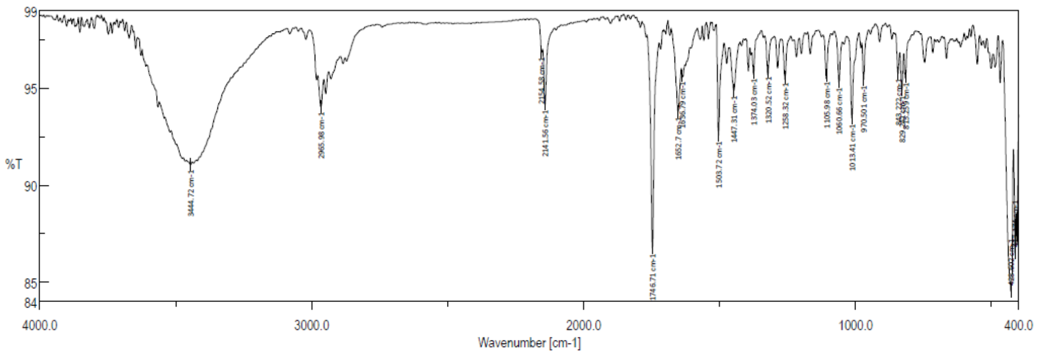

- ^1H NMR

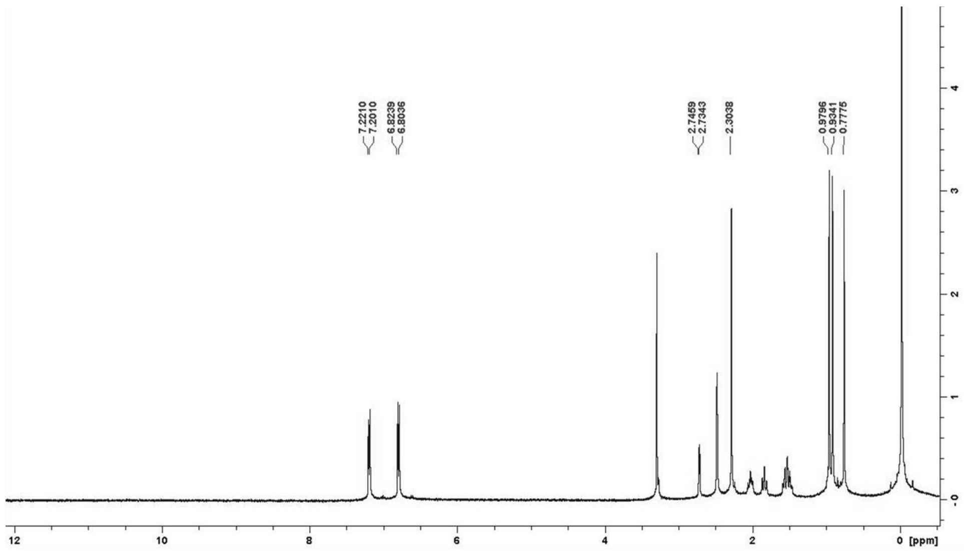

- ^{13}C NMR

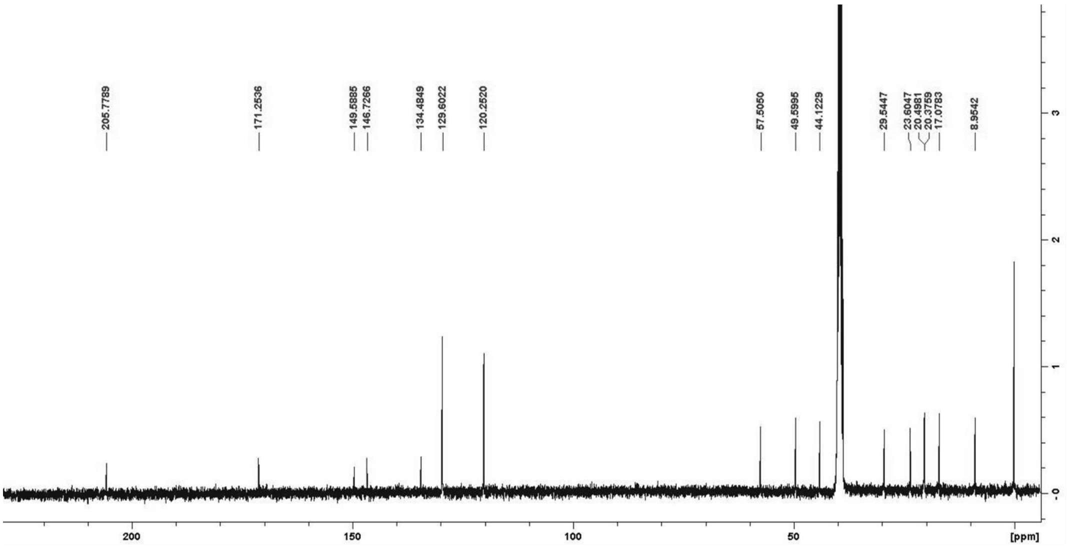

Compound **8** - $K_3[\{Au(CN)_2\}_3(OC_{10}H_{14}NC_6H_4OH\text{-}m)]$

- FTIR

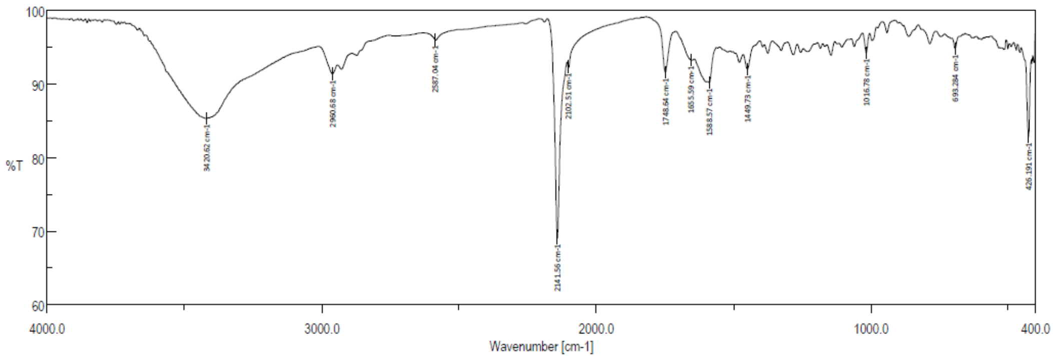

- ^{1}H NMR

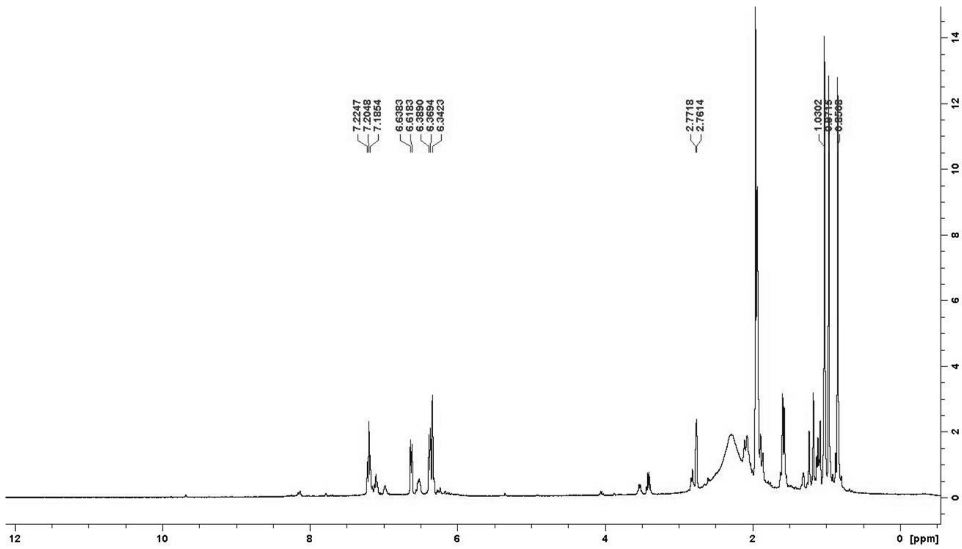

- ^{13}C NMR

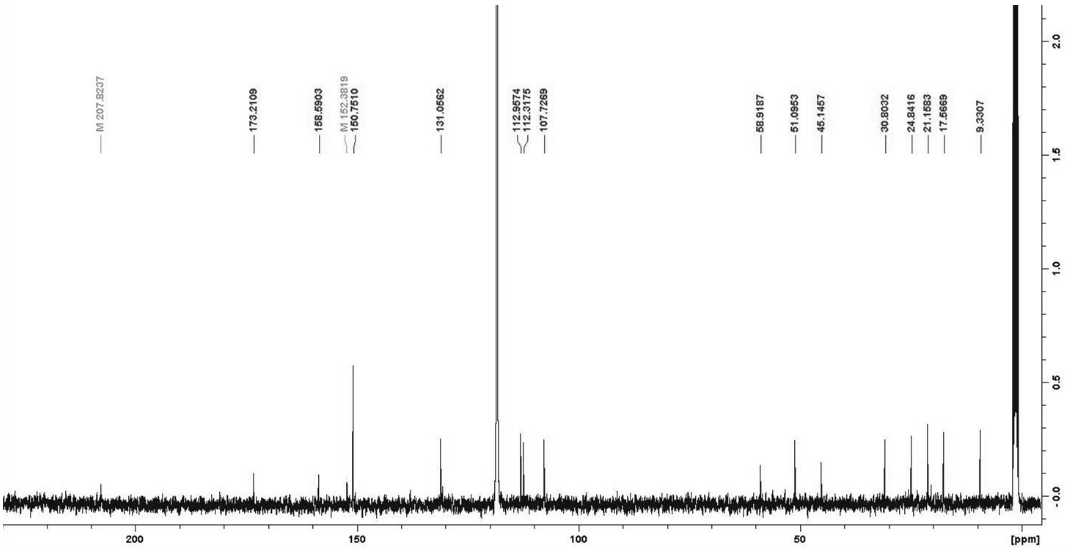

Compound **9** - K[Au(CN)$_2$(OC$_{10}$H$_{14}$NNH$_2$)]

- FTIR

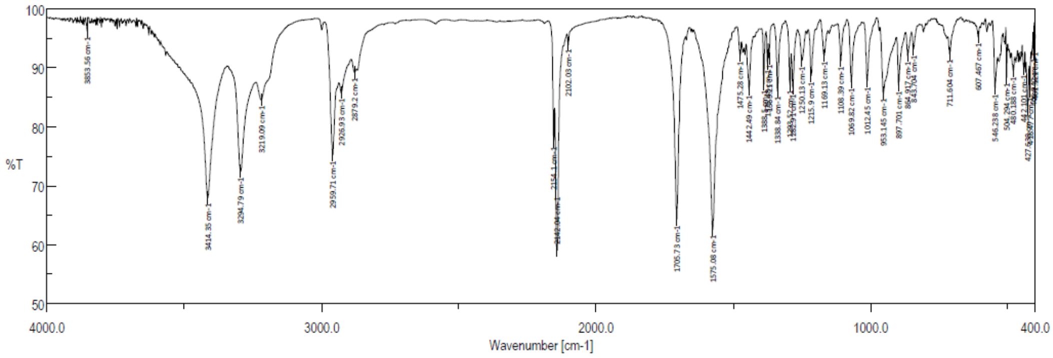

- ^{1}H NMR

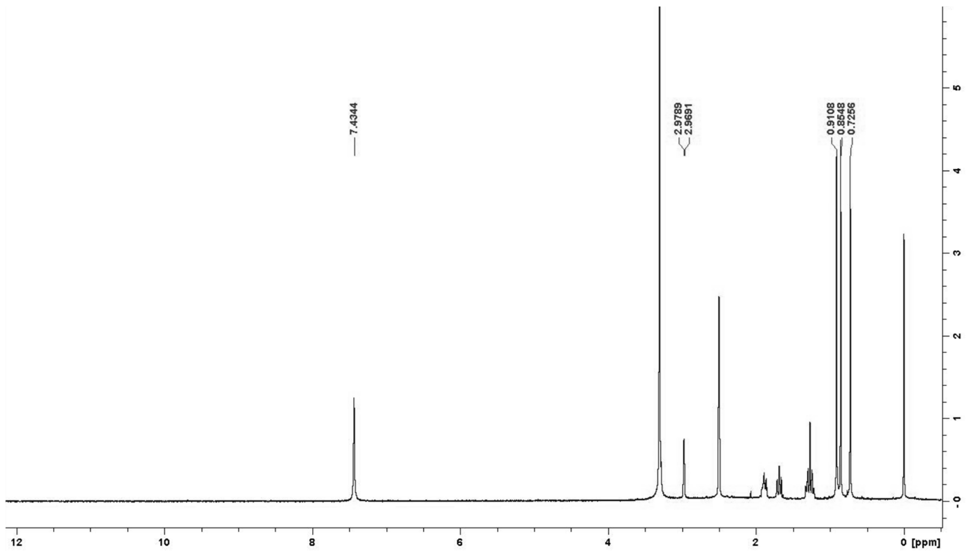

- ^{13}C NMR

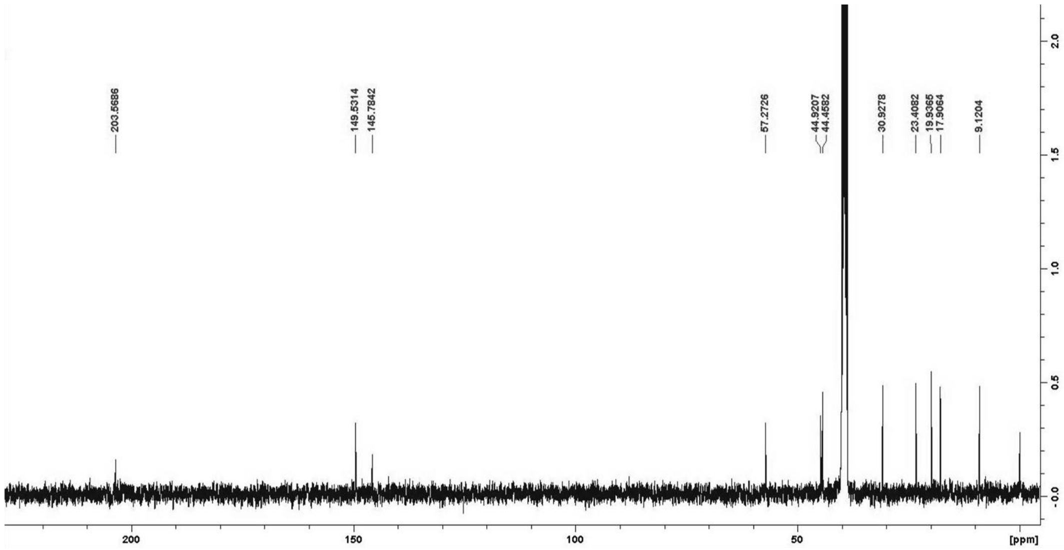

Compound **10** - K[Au(CN)$_2$(OC$_{10}$H$_{14}$NOH)$_2$]·$^1/_2$H$_2$O

- FTIR

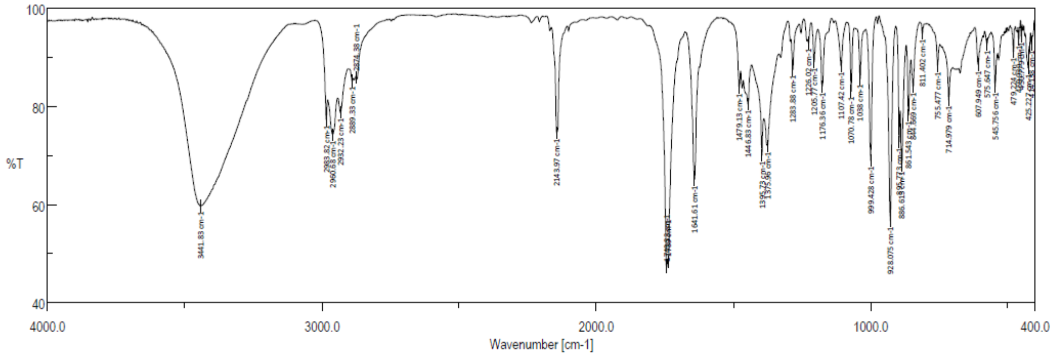

- ^{1}H NMR

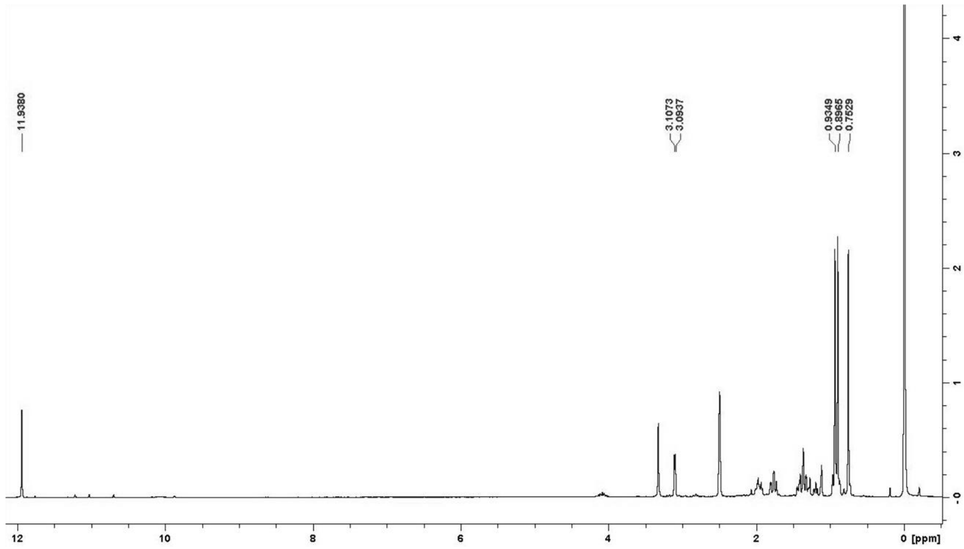

- ^{13}C NMR

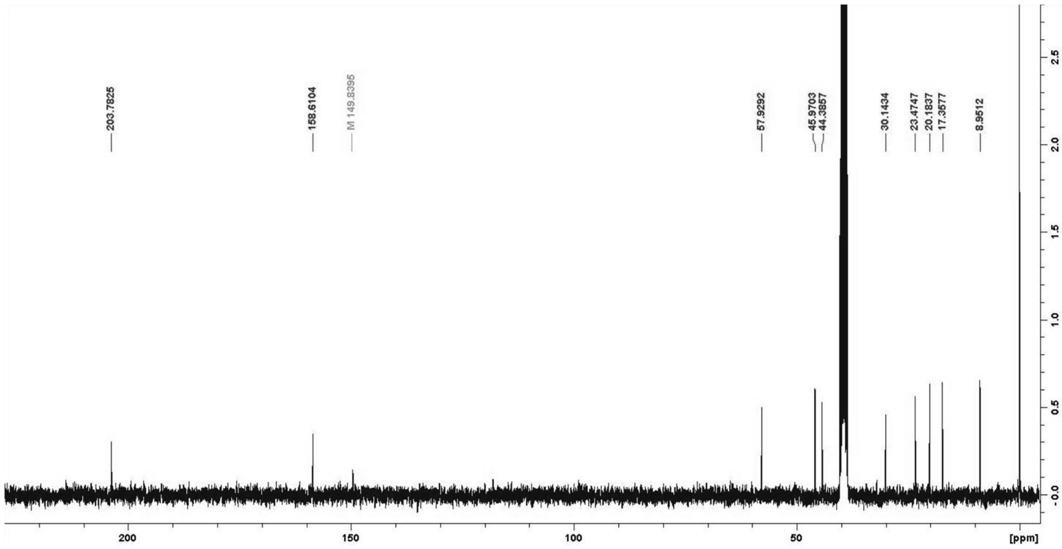

Compound **11** - $K_3[\{Au(CN)_2\}_3(C_{10}H_{14}NC_6H_4N)]\cdot H_2O$

- FTIR

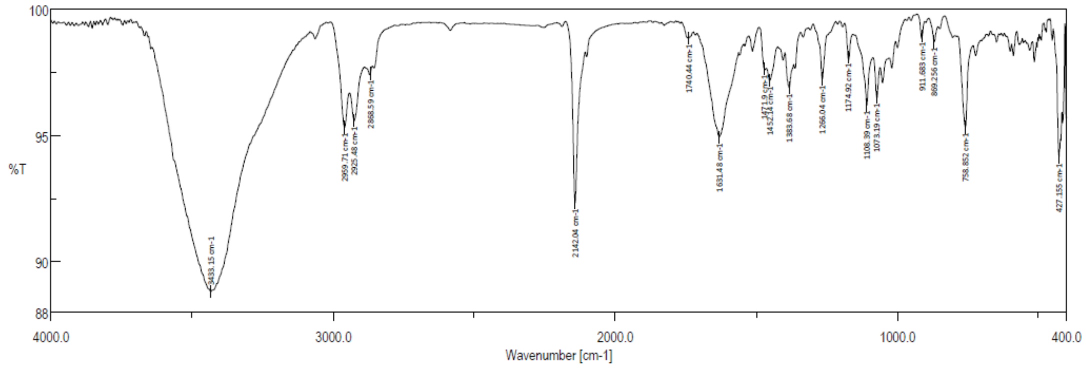

- ^{1}H NMR

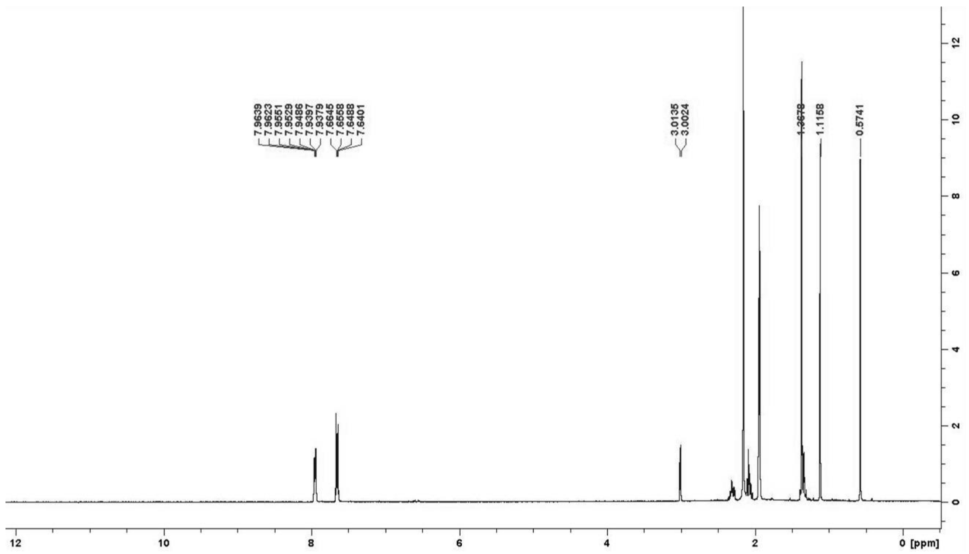

- ^{13}C NMR

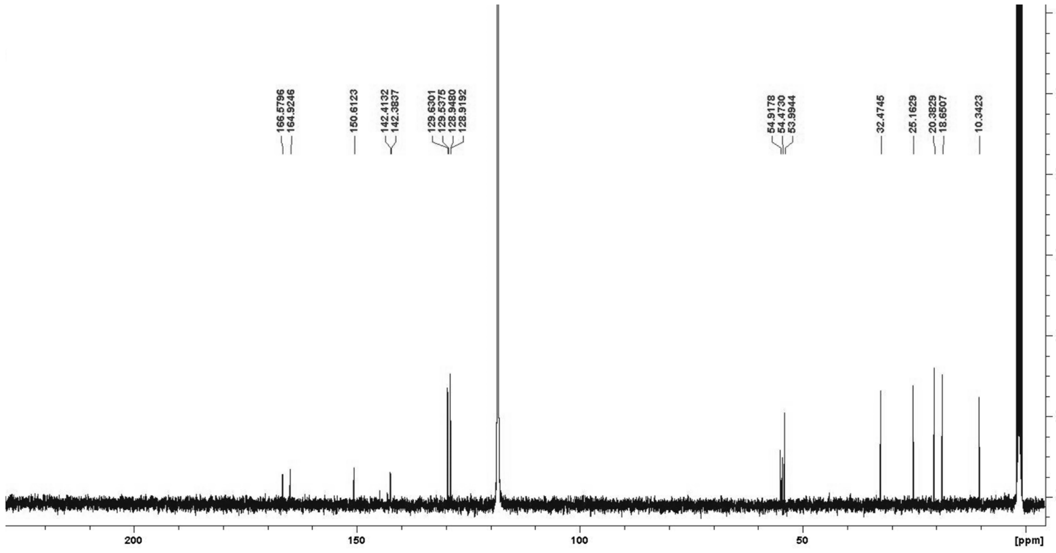

Compound **12** - K[Au(CN)$_2$(C$_{10}$H$_{14}$NC$_6$H$_4$N)$_3$]·H$_2$O

- FTIR

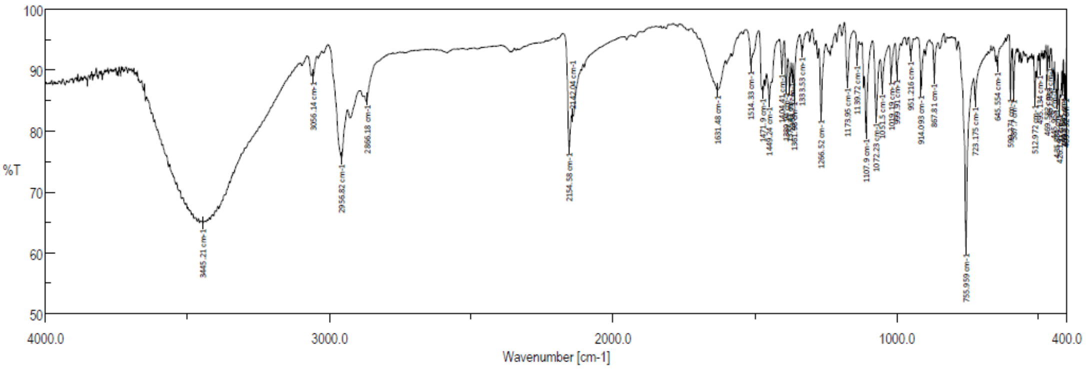

- ^{1}H NMR

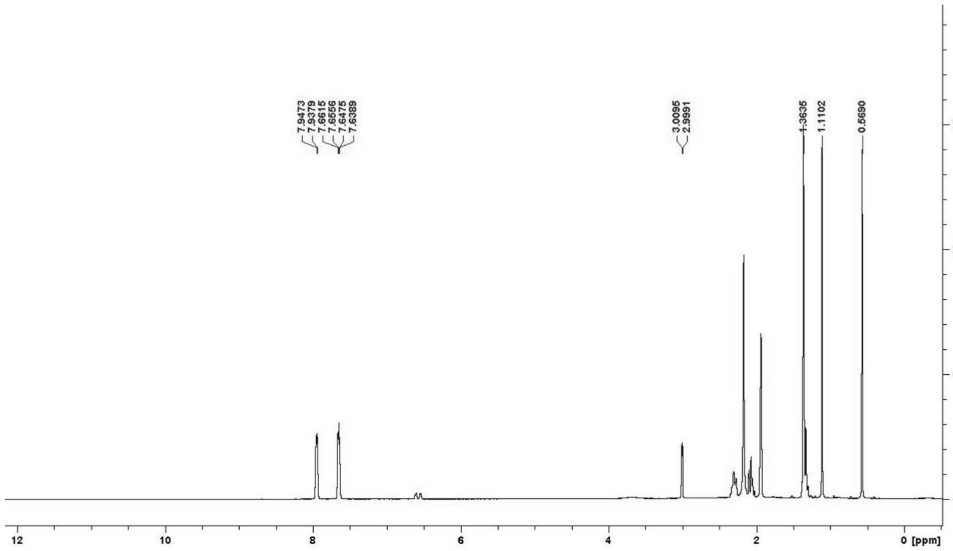

- ^{13}C NMR

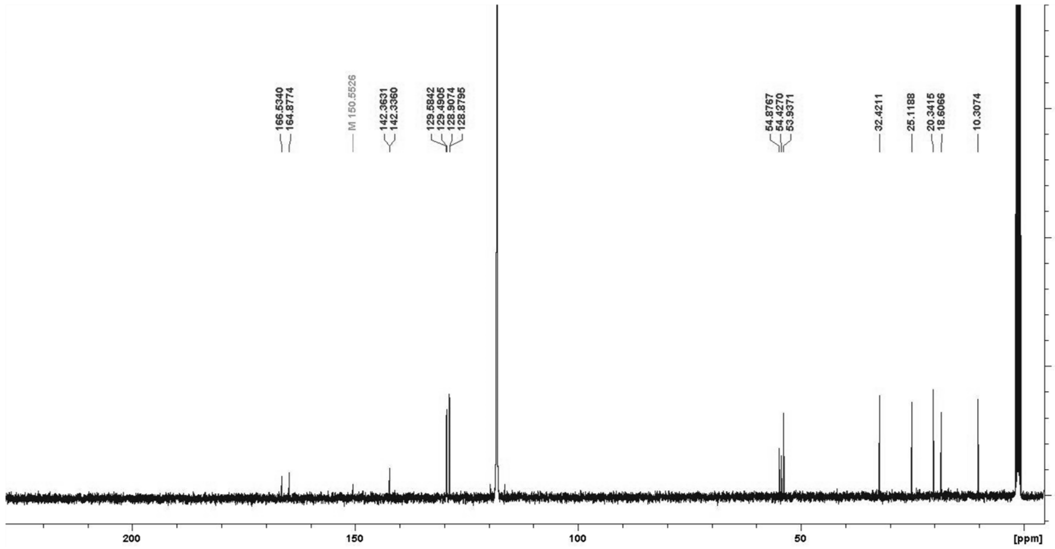

Compound **13** - $[Au(CN)\{(OC_{10}H_{14}N)_2C_6H_4\text{-}m\}]\cdot CH_3CN$

- FTIR

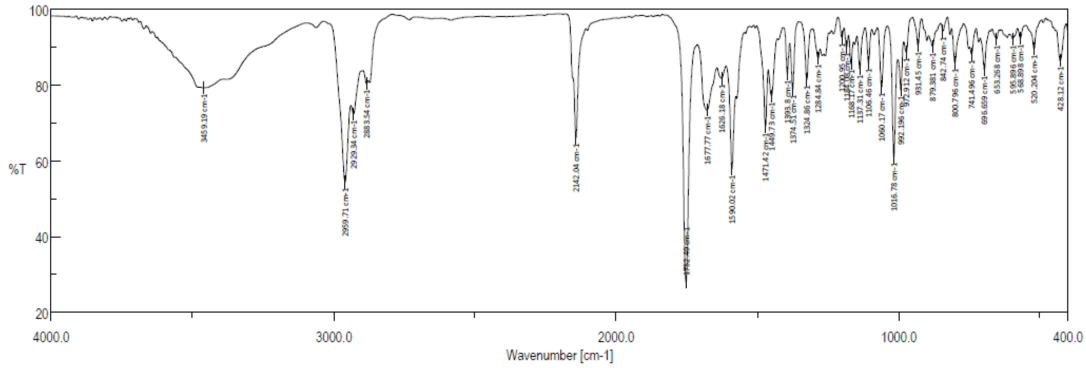

- ^{1}H NMR

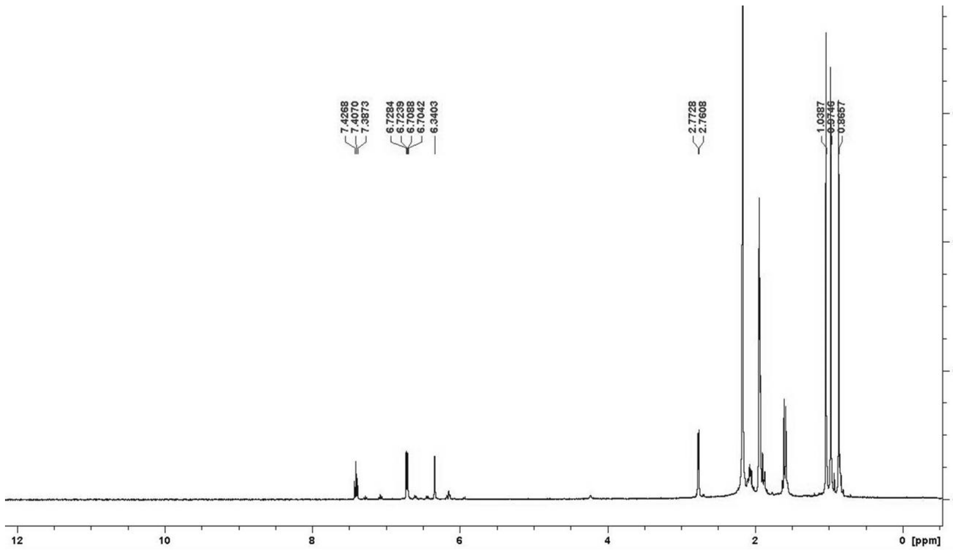

- ^{13}C NMR

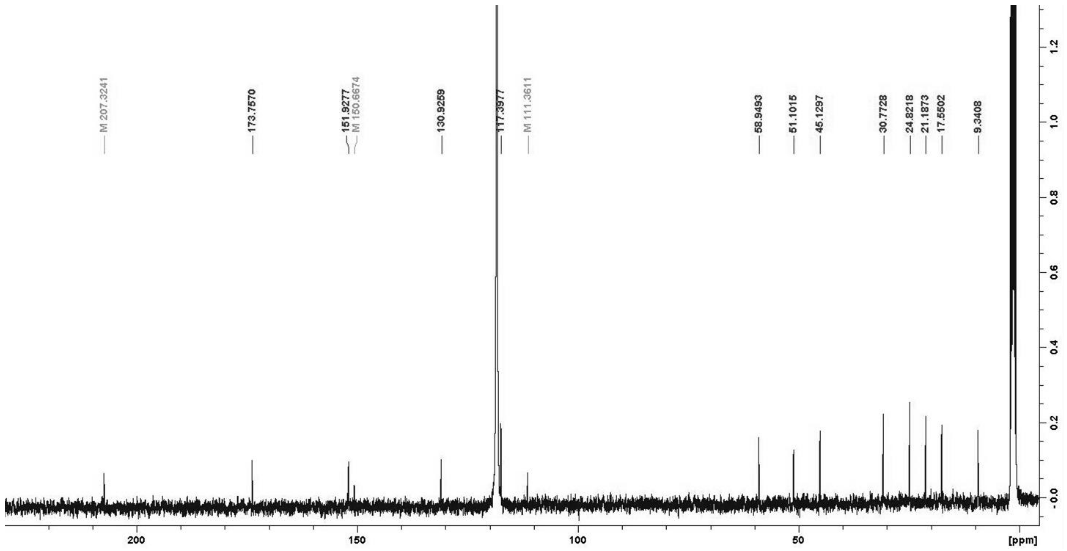

Compound **14** - $K_3[\{Au(CN)_2\}_3\{(OC_{10}H_{14}N)_2C_6H_4\text{-}p\}]\cdot 3H_2O$

- FTIR

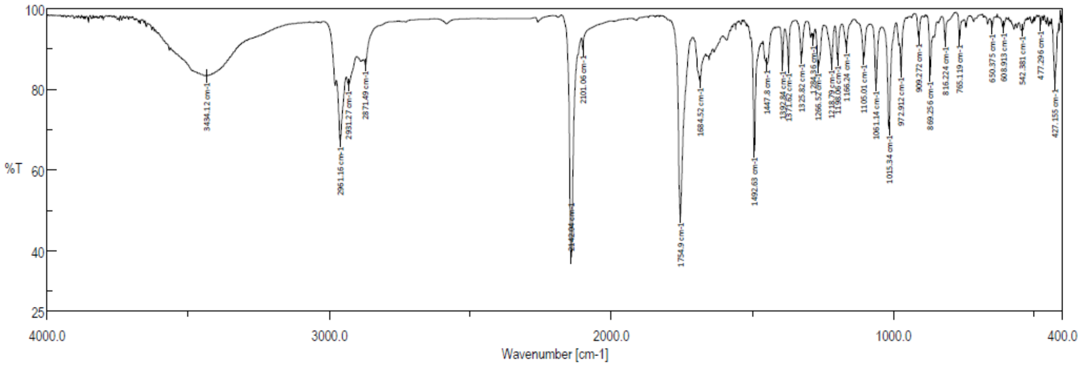

- ^1H NMR

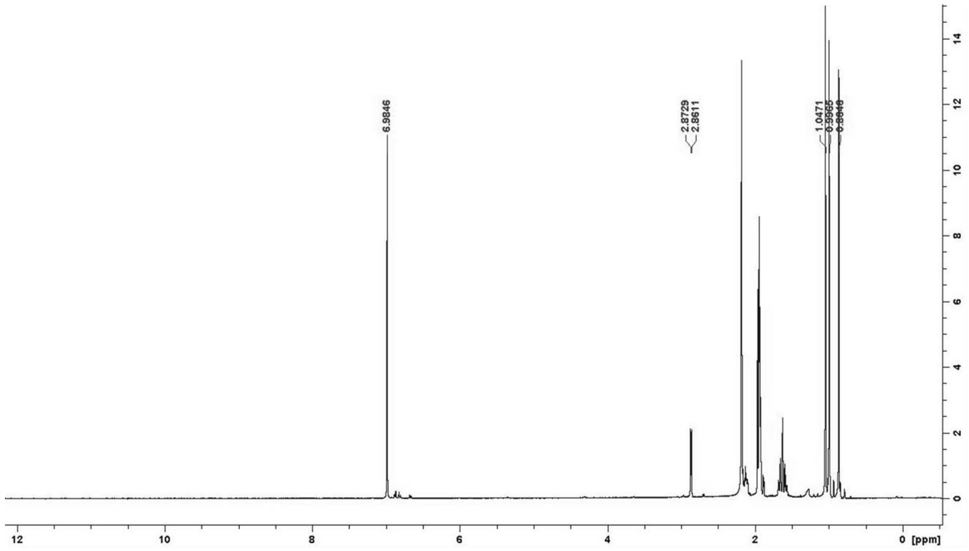

- ^{13}C NMR

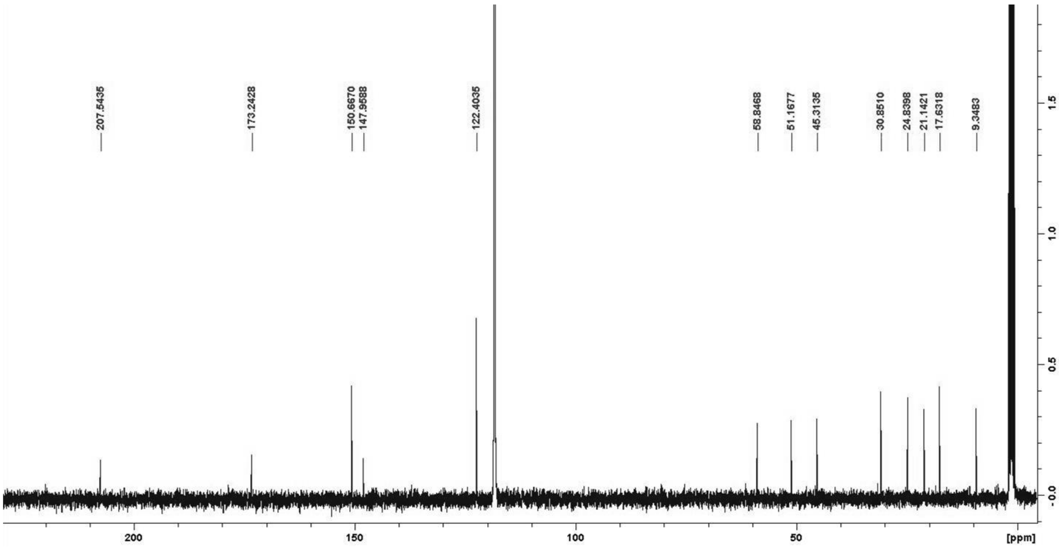

Supplement: Supplementary file 1 [file antibiotics-10-01272-s001.zip › antibiotics-1422020-supplementary.pdf]
